# Engineering Coil-Coiled Domains for the Design of Modular Theranostic Agents: Galectin‑3 as a Targeting Moiety

**DOI:** 10.1021/acs.biomac.6c00502

**Published:** 2026-06-27

**Authors:** Chiara Burgio, Maria D. Giron, Mariano Ortega-Muñoz, Daniel Lucena, Rafael Salto

**Affiliations:** † Department of Biochemistry and Molecular Biology II, School of Pharmacy, 16741 University of Granada, Granada E-18071, Spain; ‡ Department of Organic Chemistry, School of Sciences, Biotechnology Institute, University of Granada, Granada, E-18071 Spain; § Unit of Excellence in Chemistry Applied to Biomedicine and the Environment of the University of Granada, Granada E-18071, Spain

## Abstract

To generate drug-targeting agents with a modular and efficient design for disease treatments, including cancer, we used protein coil-coiled dimerization motifs to eliminate the need for chemical conjugation techniques, generating a versatile theranostic system: a targeting moiety genetically fused to an E_3_ coil and a fusion protein of maltose-binding protein and a monomeric streptavidin that includes a K_3_ coil. The system can be directed to other targets by changing the targeting moiety. The second component enables simultaneous binding to two different ligands (maltosylated or biotinylated). We tested the system using a truncated human Galectin-3 as the targeting moiety, which binds to β-galactoside-containing oligosaccharides overexpressed on the plasma membranes of tumor cells, and biotinylated doxorubicin as the cargo molecule. *In vitro* experiments using cell lines that express or do not express Galectin-3 ligands and*in vivo* mouse xenografts demonstrated the system’s capability, specificity, and flexibility.

## Graphical abstract

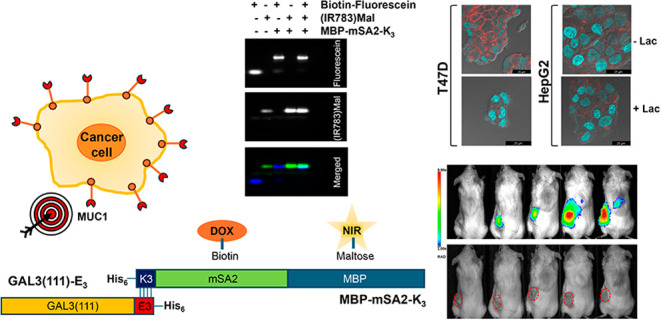

## 1 Introduction

Various components are needed to achieve a specific theranostic agent: a targeting molecule to confer specificity, a drug to provide antineoplastic activity, and a visualization agent. Furthermore, these components must be assembled into a particle suitable for *in vivo* delivery.

The targeting molecules are ligands that specifically recognize cancer cell receptors or biomarkers, initiating docking and, if possible, internalization. Many targeting molecules have been proposed, including small molecules and proteins. Examples of small molecules include carbohydrates, hyaluronic acid, aptamers, and even glycated polymers.^1^ As targeting proteins, antibodies are widely used,^2^ but other targeting proteins, such as lectins or peptidic hormones, have also been explored to a lesser extent.^3,4^

Usually, these components are combined either through direct coupling or conjugation into soluble or insoluble nanoparticles based on lipids, polymers, or even viruses. These coupling or conjugation processes must be specifically designed for each type of molecule and involve complex covalent and noncovalent methods. Efforts have been made to simplify the creation of these targeting nanoparticles, such as using monoclonal antibodies-streptavidin fusion proteins to bind biotinylated cargos for targeted delivery.^5,6^ Our research group has also proposed further steps, using single-chain variable fragments genetically fused to the maltose-binding protein and maltosylated cargo molecules.^4,7^

Coil-coiled motifs are a common structural motif and very diverse. For biotechnological uses, they can be generated by a synthetic coil protein domain (E_3_) with the sequence (EIAALEK), widely used in protein engineering for purification and dimerization.^8,9^ The sequence is designed to form a stable heterodimer with a complementary sequence, (KIAALKE) (K_3_).^10^ Although coiled-coil peptides are not as versatile as nucleic acids in terms of the number of potential complementary sequences, when assembled in the correct orientation,^10^ they have high sequence selectivity. They can be easily genetically encoded into the protein of interest.^11^ Coil-coiled motifs could be parallel or antiparallel, and both are essential tools in biotechnology. The use of coil-coiled motifs for dimerization has numerous applications in the design of biosensors, drug delivery systems, and the study of membrane receptor dimerization.^12,13^

Therefore, in this article, we expand the concept of modularity in designing drug-targeting systems by using a coiled-coil dimerization motif. One member of the delivering system contains a targeting moiety fused to an E_3_ coil, while a fusion protein of maltose-binding protein (MBP) and monomeric modified streptavidin (mSA2) and the K_3_ coil is the other partner. This second partner allows binding of two different ligands simultaneously (maltosylated or biotinylated), enabling drug transport and visualization. As proof of concept, we have used a human lectin (Galectin-3), which recognizes glycosylated proteins on the surface of cancer cells^14^ as the targeting molecule, and biotinylated doxorubicin or maltosylated ligands as the cargo molecules. *In vitro*, in cells, and in animal model experiments, our results support the use of this strategy to target cells expressing glycosylated plasma membrane proteins recognized by Galectin-3 to deliver biotinylated doxorubicin or maltosylated near-infrared fluorophores,^7^ generating a theranostic agent. Furthermore, by changing the first partner as the targeting moiety, the system can be directed to a variety of cell membrane targets, supporting the system’s versatility.

## 2 Experimental Section

### 2.1 Cloning, Expression, and Purification of Recombinant Proteins

The coding sequence of the human Galectin-3 (GenBank #AB006780) was amplified by PCR using the oligonucleotides Gal3-f and Gal3-r and retrotranscripted RNA from U-937 cells (ATCC CRL-1593.2) as a template. The sequences of these oligonucleotides (described in Supporting Information) also include *BamH*I and *Sal*I restriction sites, respectively. In addition, a fragment of the coding sequence of the human Gal-3, corresponding to the carbohydrate recognition domain and termed Gal-3(111), was also amplified using a Gal-3(111)-f oligonucleotide that includes a *BamH*I restriction site. PCR fragments were subcloned into pMAL-TEV-His vector, a modified pMAL-c2X expression vector (New England Biolabs, Ipswich, MA, USA) where a TEV protease cleavage site has replaced the factor Xa cleavage sequence, and also encodes for an additional poly-His tail,^7^ to generate pMAL-TEV-Gal3-His and pMAL-TEV-Gal-3(111)-His plasmid, respectively.

For the construction of a coil-coiled-based drug delivery system, a flexible linker (coding for (GGGGS)_2_) and a linker coding for an E_3_ coil motif^15^ were cloned downstream of the Gal-3(111) coding sequence, rendering the pMAL-TEV-Gal-3(111)-E_3_-His plasmid.

The coding sequence for a monomeric avidin mSA2,^16^ including a flexible linker, was amplified by PCR from plasmid pET-MBP-mSA2 (a gift from Sheldon Park, Addgene plasmid #52319) and then, together with a linker coding for a K_3_ coil motif^15,17^ were cloned into the pMAL-TEV-His vector to render the pMAL-TEV-mSA2-K_3_-His plasmid. The sequences of the plasmids used in this study are included in the Supporting Information.

For the expression of the different recombinant proteins, plasmids were transformed in Novagen’s Rosetta Blue competent cells (Merck, Madrid, Spain) and grown in Luria–Bertani Broth medium with appropriate antibiotics at 37 °C. Cells were incubated until an OD_600_ = 0.5 was reached, and then, the expression of the recombinant proteins was induced by the addition of IPTG (0.5 mM final concentration). Cells were grown at 30 °C for an additional 8 h, and then the bacterial pellet was harvested and stored at −20 °C.

For the purification of the recombinant proteins, bacterial pellets were resuspended in 20 mM HEPES, 500 mM NaCl, pH 8.0 buffer and sonicated. The lysed bacterial suspension was centrifuged for 30 min at 12000 × g to remove cell debris and the supernatant was filtered through a 0.45 μm filter. Supernatants from pMAL-TEV-Gal-3-His and pMAL-TEV-Gal-3(111)-His transformed bacteria were incubated overnight at 10 °C with a modified version of TEV protease with improved stability (TEV S219V)^18^ to process the fusion protein. The supernatant from pMAL-TEV-mSA2-K_3_-His transformed bacteria was not digested. Supernatants were loaded into a HisTrap HP 1 mL column (GE Healthcare Life Sciences, Chicago, IL, USA) equilibrated in 20 mM HEPES, 20 mM imidazole, pH 8.0 buffer. After washing, proteins were eluted using 20 mM HEPES, 500 mM imidazole, pH 8.0, and the proteins were concentrated and buffer exchanged to PBS by centrifugation at 10000 × g using a PES (poly­(ether sulfone)) Centrifugal filter (VWR International, Barcelona, Spain). Protein concentration was measured by the BCA method. The purity of the proteins was confirmed by SDS-PAGE electrophoresis (Figure S1, Supporting Information).

### 2.2 Analysis of the Ligand Binding to MBP-TEV-mSA2-K_3_ and the Association of Recombinant Coil-Coiled Proteins

The binding of Biotin-fluorescein or (IR783)­Mal^7^ to MBP-TEV-mSA2-K_3_ has been detected by agarose gel electrophoresis (0.8% w/v) using TAE buffer (40 mM, Tris-acetate, 1 mM EDTA). Ligands were preincubated for 30 min with MBP-mSA2-K_3_ protein and then analyzed by agarose electrophoresis. Gels were visualized using a ChemiDoc MP Imaging System (Bio-Rad, Madrid, Spain) using a fluorescein filter for the detection of Biotin-fluorescein and an Alexa680 filter for the detection of (IR783)­Mal. The binding of the ligands to the protein was detected by changes in fluorescence intensity upon binding to the protein (IR783)­Mal, or changes in the electrophoretic mobility (Biotin-fluorescein).

The association of the Gal-3(111)-E_3_ and MBP-TEV-mSA2-K_3_ proteins was analyzed by agarose gel electrophoresis (0.8% w/v) using TAE buffer (40 mM, Tris-acetate, 1 mM EDTA) containing 5 μL/mL trichloroethane. After electrophoresis, proteins were detected using a UV light-induced reaction between the Trp present in the protein and trichloroethane to produce a fluorescent adduct,^19^ as described in the ChemiDoc MP stain-free protocol. Changes in the electrophoretic mobility detected the protein association.

### 2.3 Synthesis of Biotin-Doxorubicin (b-DOX)

b-DOX (Figure 1D) was synthesized as described in Figure S2, Supporting Information, by first reducing D-biotin to D-biotinol, to generate the D-biotinyl vinylsulfonate. This molecule has been conjugated with doxorubicin through an aza-Michael reaction to generate b-DOX.

**Figure 1:**
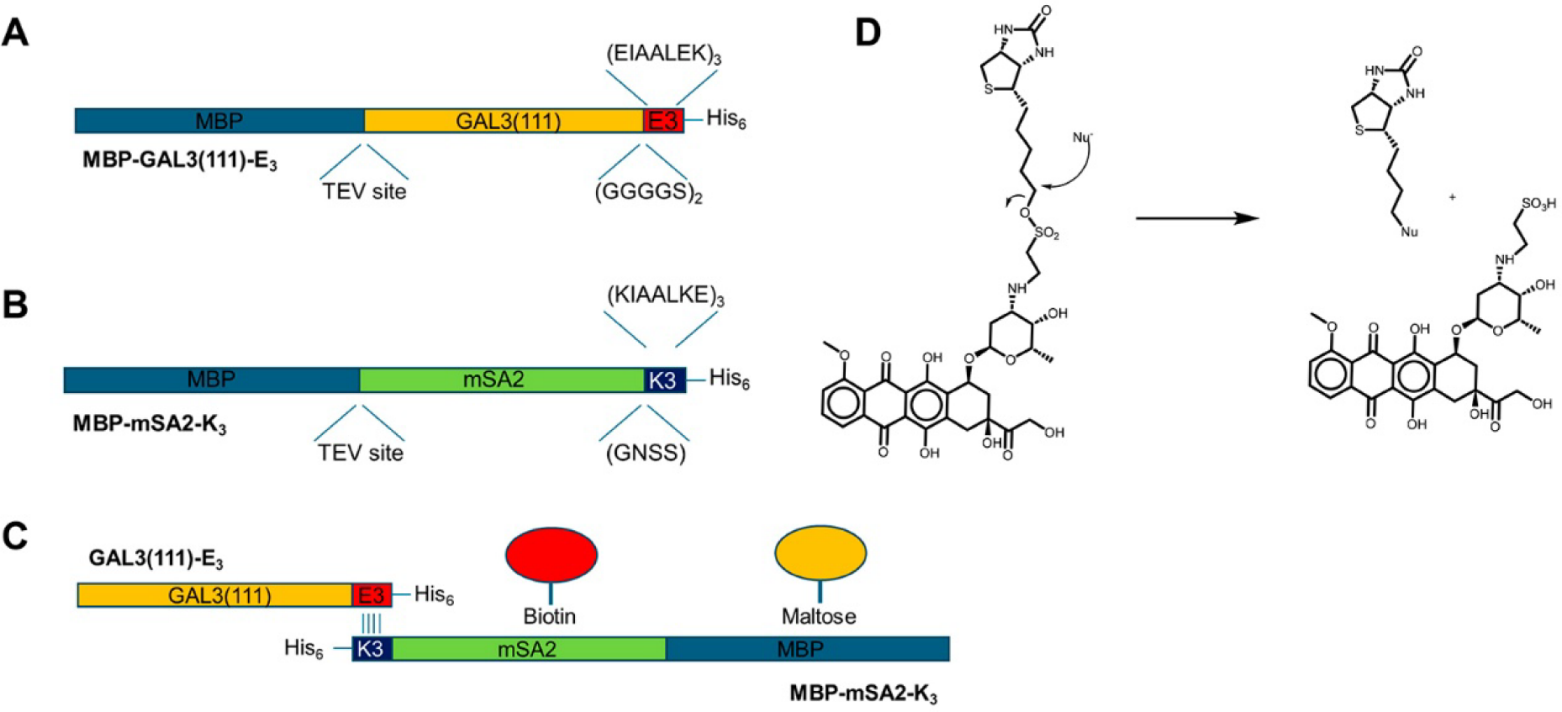
Scheme of the recombinant protein and ligands used to design a coil-coiled theranostic delivery system. (A), (B) Scheme of the recombinant proteins used in the study. (C) Proposed assembly of the recombinant proteins after TEV cleavage of the MBP-GAL-3(111)-E_3_ protein. (D) Biotinylated doxorubicin (b-DOX) used as a ligand and proposed intracellular cleavage produced by nucleophilic reducing agents.

### 2.4 Human Cell Lines

Cervix adenocarcinoma HeLa cells (ATCC CCL-2), Breast ductal carcinoma T47D cells (ATCC HTB-133), Breast gland adenocarcinoma MCF7 cells (ATCC HTB-22), and hepatocellular carcinoma HepG2 cells (ATCC HB-8065) were grown at 37 °C in Dulbecco’s Modified Eagle’s Medium (DMEM) supplemented with 10% (v/v) fetal bovine serum (FBS), 2 mM glutamine plus 100U/mL penicillin, in an atmosphere of 5% CO_2_ and 95% air and maintained at subconfluent densities in the growth media. These cell lines have been used in an authorized cell culture laboratory (Junta de Andalucía, Spain, authorization code A/ES/19/I-39).

### 2.5 Inactivation of Human MUC1 by CRISPR-Cas9

Human MUC1-silenced cells were generated using a guide RNA (gRNA) for inducing a double-strand break at the exon 1 of the human MUC1 gene: 5′-aagaaaggagactgggtgcc-3′, where the PAM sequence is underlined. The sequence was designed using https://chopchop.cbu.uib.no/.^20^ The gRNA without the PAM sequence was cloned into the CRISPR-Cas9 plasmid PX459 (pSpCas9­(BB)-2A-Puro, Addgene #62988)^21^ to render plasmid PX459-MUC1.

HeLa cells were transfected with the PX459-MUC1 using Lipofectamine 2000. The transfected cells were grown for 2 days and then selected with puromycin (Thermo-Fisher, Waltham, Massachusetts, USA) for an additional 2 days. The surviving cells were clonally diluted into 96-well plates to generate clonal populations from single cells.

Western blotting was used to analyze isolated clones using an anti-MUC1 antibody (Mucin 1/MUC1 Antibody (VU4H5) (Santa Cruz Biotechnology, Dallas, Texas, USA, Cat. sc-7313) (Figure S3, Supporting Information). Furthermore, genomic DNA was isolated from wild-type cells and positive clones to PCR amplify the sequence flanking the indel introduced, and the silencing was confirmed by Sanger sequencing.^22^ Two silenced clones were pooled and used for experiments.

### 2.6 Western Blotting

Whole-cell extracts from HeLa cells were lysed using RIPA buffer containing protease and phosphatase inhibitors, followed by SDS–PAGE analysis. Anti-AKT (Cat. #4691S, CST), antiphospho-AKT (Ser473) (Cat. #9271T, CST) antibodies were used. Immunoblots were developed using StarBright Dye Secondary Antibodies (Bio-Rad, Madrid, Spain) in a ChemiDoc MP Imaging System. Quantification of the bands was performed using the ImageLab v6.0.1 Software (Bio-Rad, Madrid, Spain).

### 2.7 Study of the Cell Uptake

A total of 1.6 × 10^5^ cells/well were seeded in 48-well plates and grown for 24 h. Cells were incubated with Doxorubicin (DOX), biotin-ATTO647 (ATTO-TEC GmbH, Siegen, Germany), biotin-Doxorubicin (b-DOX) alone or bound to monomeric streptavidin (mSA2) (Sigma, Madrid, Spain) or the recombinant proteins described in the article. Cell uptake was measured and expressed as fluorescence per mg of protein.

For studies using biotin-ATTO-647 or b-DOX, these were incubated with different proteins, including mSA2, Gal-3(111)-E_3_ and maltose-binding protein linked to mSA2 and K_3_ (MBP-mSA2-K_3_). The binding of Gal-3(111)-E_3_ and MBP-mSA2-K_3_ was performed by mixing for 15 min before adding them to the cells. In the case of preincubation with lactose, 2 mg/mL lactose was added once the targeting molecule was formed, and the mixture was incubated for 30 min before being added to the cells.

The fluorescence spectra were captured at maximum excitation (λex) and emission (λem) wavelengths, being 480 and 520 nm for fluorescein, 470 and 560 nm for doxorubicin and 646 and 666 nm for ATTO-647.

### 2.8 Cell Toxicity Assay

A total of 1.6 × 10^5^ cells/well were seeded in 48-well plates and cultured for 48 h in the presence of the different compounds. In this case, DOX or biotin-DOX were used. Proliferation was measured using the 3-(4,5-dimethylthiazol-2-yl)-2,5-diphenyl-2H-tetrazolium bromide method. The results were expressed as the percentage of cell toxicity based on the 0-h untreated cells.

### 2.9 Confocal Microscopy

For confocal cell analysis, cells were seeded on glass coverslips and incubated for 2 h with the same molecules described in the cell uptake section. The coverslips were mounted on glass slides using ProLong Gold Antifade Reagent (Thermo Fisher Scientific, Waltham, Massachusetts, United States). Confocal microscopy was performed using a Leica TCS-SP5 II multiphoton confocal microscope (Central Facilities, University of Granada, Spain), with a sequential acquisition mode to separately collect images in individual channels for color analysis. A pinhole of 1 Airy unit was utilized. Images were acquired at a resolution of 1024 Å. Series were captured in the xyz mode. Data was processed using the Leica Application Suite software package.

### 2.10 *In Vivo* Studies

Male NSG immunodeficient mice (6–8 weeks of age, 25–30 g weight) were purchased from the Animal Facility at the University of Granada and maintained in accordance with guidelines established by Directive 2012/707/UE and the approval of the Committee on Animal Research at the University of Granada (07/03/2022/025). For xenograft models, HeLa cells were trypsinized and resuspended in PBS (density equal to 2 × 10^7^ cells/mL). Cells (1 × 10^6^) were subcutaneously inoculated into the left dorsal region of mice. When tumor sizes reached 1 to 6 mm in diameter, mice were injected in the tail vein with the different targeted antitumor therapeutics bound to Biotin-ATTO740 (ATTO-TEC GmbH, Siegen, Germany). The *in vivo* imaging over time assays were performed in an IVIS Spectrum (Caliper Life Sciences, MA, USA). Isoflurane-anesthetized mice were placed in the dark chamber for fluorescence (excitation/emission, 740/760 nm) acquisition. Images were taken and analyzed with the Aura Image 4.5 software package (Spectral Instruments Imaging, Tucson, AZ, USA).

### 2.11 Statistical Analysis

One-way or two-way analysis of variance statistical analysis was performed using GraphPad Prism 8.0.2 software (San Diego, CA) to evaluate the significance of the experimental data. A p-value of 0.05 was selected as the significance level.

## 3 Results and Discussion

Protein dimerization plays a central role in biology. In addition to its function in physiological and pathological processes, the use or the modification of naturally occurring dimerization motifs has been exploited in Biology and Biotechnology. These motifs have been incorporated into proteins to study cell trafficking and other cellular processes^23^ or, for example, for affinity chromatography-based protein purification.^24^

One of the best-characterized dimerization motifs is a two-stranded α-helical coiled coil composed of repeated seven amino acid units that contain spaced hydrophobic residues. Ionic interactions further stabilized the structure due to the presence of polar and charged residues. An example of this structure is a coiled-coil pair (E_3_/K_3_).^8,25^ This pair has only 21 amino acids in length and provides a highly stable dimerization motif with a 1:1 stoichiometry at nanomolar affinity.^15^

In this article (Figure 1C), we propose a modular, versatile system as a theranostic agent that employs this dimerization motif. One component of the system includes a targeting moiety (a truncated version of human Galectin-3) genetically fused to the E_3_ coil, while the other component, which bears the K_3_ coil domain, consists of a fusion protein between maltose-binding protein (MBP) and monomeric modified streptavidin (mSA2). This second component enables the binding of two different ligands simultaneously (maltosylated or biotinylated), facilitating drug transport and visualization. Additionally, by replacing the first component with a different targeting moiety, the system can be directed to various cell membrane targets, demonstrating its versatility.

Cell surface interactions modulate physiological and pathological processes. Some are involved in cancer development and metastasis and, therefore, have been extensively studied to block these processes. Altered glycosylation of plasma membrane proteins is common in cancer. One of these proteins, MUC1, a member of the mucin family of proteins, is overexpressed in several tumors. Also, it shows abnormal glycosylation patterns, including the presence of the Thomsen-Friedenreich antigen (a Galβ1–3GalNAc disaccharide).^26^ LAMP1 is another highly glycosylated lysosomal protein that is overexpressed on the plasma membrane of cancer cells^27^ and may act as a ligand for lectins.

LAMP1 ^28^ and MUC1 ^26^ carbohydrate moieties are recognized by Galectin-3 (Gal-3), a member of the family of animal lectins able to bind β-galactoside-containing oligosaccharides. Gal-3 is a chimera-type galectin with a single carbohydrate recognition domain and an N-terminal domain.^29^ Interestingly, Gal-3, despite lacking a signal peptide sequence, is secreted outside the cell.^30^ The interaction of the secreted Gal-3 with MUC1 at the plasma membrane, through stimulation of the Akt signaling pathway, can accelerate tumor progression.^14^ On the contrary, in the brain, the blockade of Gal-3 expression restrains cancer growth^31^ and, in concordance, antagonists of Gal-3 binding, such as GS-100, have been proposed as promising cancer therapeutics.^32^

An important feature of Gal-3 is its modular domain. While its C-terminal carbohydrate recognition domain is sufficient to bind to galactose-bearing proteins,^33^ the N-terminal domain is required to increase the multivalence of the recognition and to elicit the intracellular activation of signaling pathways.^14^ Another important feature of Gal-3 is that upon binding to the galactose-containing membrane proteins, only the presence of the Gal-3 carbohydrate recognition domain in the recognition complex is needed to drive its internalization into the cell.^33^

All the above make Gal-3 an interesting target for directing drugs to cells that overexpress galactose-bearing membrane proteins such as MUC1. Moreover, the possibility of using only the carbohydrate recognition domain of Gal-3 for targeting and internalization is interesting because it allows reducing the size of the targeting protein, decreasing the immune response, and promoting the bioavailability of the theranostic agent.

First, we have compared the ability of Gal-3 or a truncated form of Gal-3 containing only the carbohydrate recognition domain to target cells bearing galactosylated ligands at the plasma membrane. To do that, we have designed a bacterial recombinant expression system to express and purify the human carbohydrate recognition domain of Gal-3 (111 amino acids), which we have termed Gal-3(111) or the complete Gal-3 lectin (Figure S1, Supporting Information). The recombinant protein has then been labeled using fluorescein, and its ability to bind to HeLa cells that overexpress MUC1 has been assessed by confocal microscopy and quantified by fluorescence measurements (Figure 2). Furthermore, to test MUC1 dependence on the uptake of the labeled proteins, MUC1 expression was silenced in HeLa cells using CRISPR-Cas9 technology (Figure S3, Supporting Information). Our results indicate that although both proteins retain the carbohydrate-binding domain, Gal-3(111)-FITC shows higher uptake compared to Gal-3-FITC in the wild-type HeLa cells, which express MUC1. When the uptake was analyzed in the silenced HeLa ΔMUC1 cells, a significantly lower uptake was detected for both proteins, supporting the idea of the specificity of the MUC1-Gal-3 interaction for ligand recognition.

**Figure 2:**
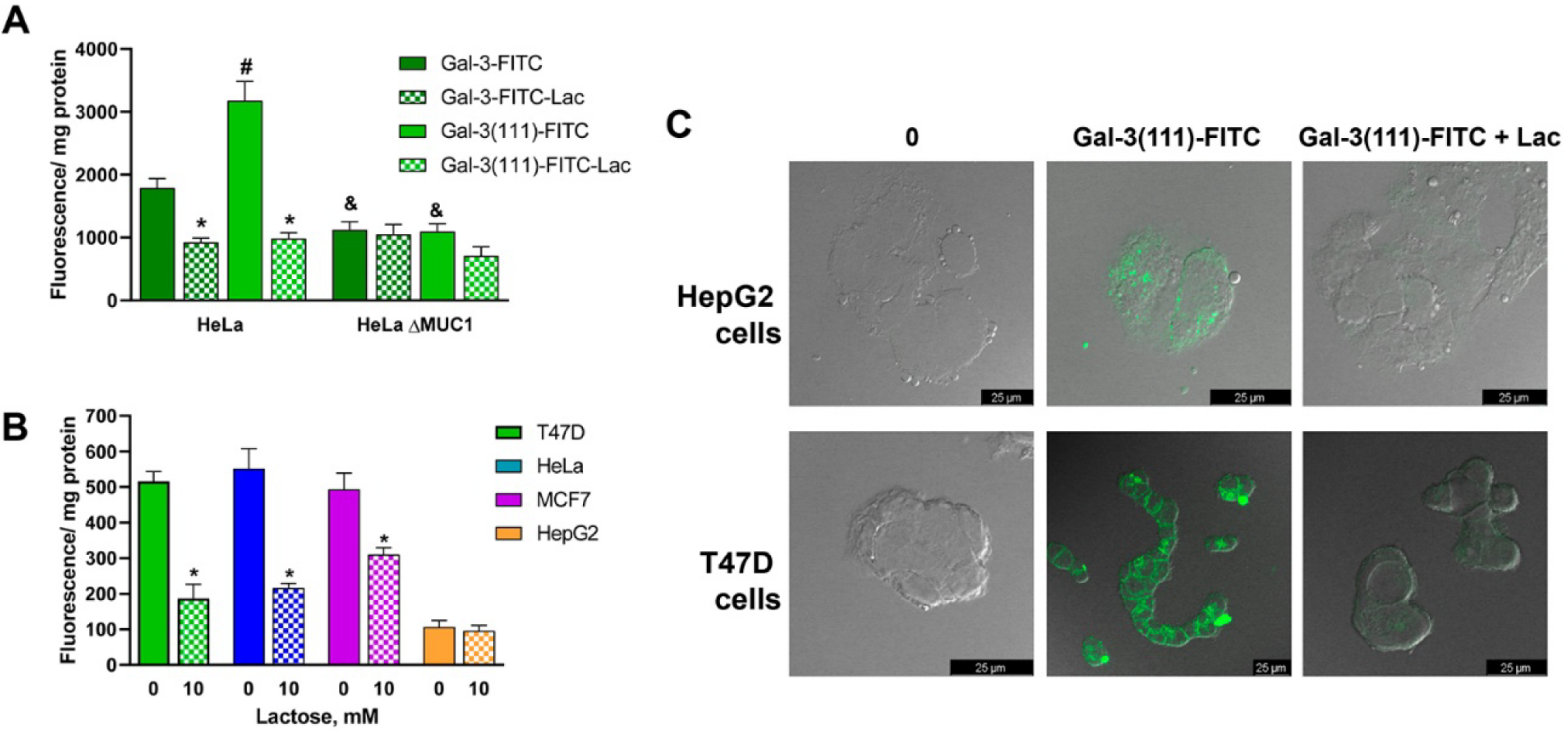
Cell binding of labeled Gal-3 or Gal-3(111) recombinant proteins. (A) Uptake of 2 mmol of the FITC-labeled proteins in the absence or presence of lactose (10 mM) in HeLa and HeLa ΔMUC1 cells. The error bars represent SEM of six replicates. *P < 0.05 vs FITC-labeled protein incubated cells; #*p* < 0.05 vs Gal-3-FITC incubated cells; & *p* < 0.05 vs FITC-labeled protein incubated HeLa cells. (B) Uptake of Gal-3(111)-FITC (2 nmol) in MUC1-positive cells (T47D, HeLa, and MCF7) and negative cells (HepG2) in the absence or presence of lactose (10 mM). The error bars represent SEM of six replicates. *P < 0.05 vs untreated cells. (C) Confocal images of cells incubated with Gal-3(111)-FITC (2 nmol) in the absence or presence of lactose (10 mM).

Since in the Gal-3(111) protein, the carbohydrate recognition domain can be blocked by incubation with 10 mM lactose, a competitor of its galactosylated natural ligands;^33^ the specificity of the binding in the presence of lactose has been assayed. In cells that express MUC1 (HeLa, T47D, MCF7), a higher binding is detected, which is mainly blocked by lactose. In contrast, in cells that do not overexpress MUC1 (HepG2), Gal-3(111) binding is lower and independent of the presence of lactose. Finally, the results have been confirmed in the positive T47D and negative HepG2 cell lines by confocal microscopy (Figure 2C), validating the use of the Gal-3(111) truncated protein as a targeting molecule for cells expressing β-galactosylated oligosaccharides at the plasma membrane.

Furthermore, while the interaction of the full-length Gal-3 protein with MUC1 at the plasma membrane, mainly through activation of Akt-dependent signaling pathways, accelerates tumor progression,^14^ the use of the truncated form Gal-3(111) could prevent this unwanted effect. We have assayed how incubation with Gal-3 or Gal-3(111) in HeLa cells influences the phosphorylative state of Akt (Figure 3). Indeed, cells incubated with Gal-3 show greater Akt activation, while Gal-3(111) does not modify Akt phosphorylation. Furthermore, Akt activation due to incubation with Gal-3 or Gal-3(111) is blocked in cells with MUC1 silenced.

**Figure 3:**
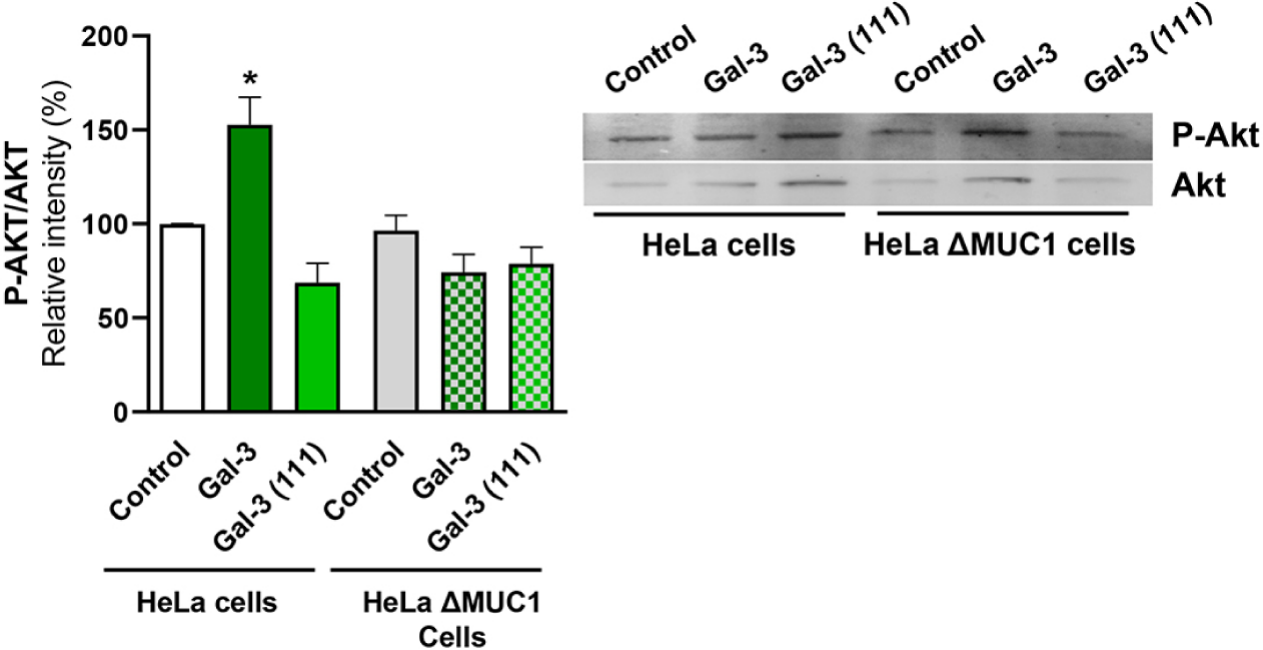
Activation of Akt after incubation with Gal-3 or Gal-3(111) recombinant proteins. HeLa or HeLa ΔMUC1 cells were incubated with Gal-3 or Gal-3(111) recombinant proteins (2 mmol) for 30 min. The phosphorylation of Akt was measured by Western blot using specific antibodies. The error bars represent SEM of four replicates. *P < 0.05 vs untreated cells.

Once it was established that Gal-3(111) can serve as a directing molecule to target specific cells without unwanted side effects and higher binding to the plasma membrane, we developed a modular system that uses a coiled-coil pair to promote dimerization. While the first partner responsible for targeting is Gal-3(111), the second member is responsible for the transport and delivery of the bioactive molecules, as well as the visualization of the complex in cell and animal models (Figure 1). With this modular design, by changing the targeting moiety of the pair, for example, for a single-chain antibody, it would be possible to direct the complex to other molecular targets without any additional optimization.

As a delivery moiety, we have selected a fusion protein between maltose-binding protein (MBP) and a monomeric modified avidin (mSA2). The MBP moiety enhances the solubility of the fusion protein and enables binding fluorescent maltosylated ligands, such as Maltose-IR783, allowing visualization of the fusion protein in biological systems.^7^ At the same time, the mSA2 moiety enables the binding of biotinylated cargos for drug delivery.

To validate this strategy, we have exploited the presence of the MBP domain in the K_3_-mSA2-MBP to express and purify the fusion protein in bacteria. The use of fusion with the MBP has been previously described to enhance the solubility of mSA2;^16^ however, these authors used a purification protocol that involves removing the mSA2-bound biotin and using high temperatures to precipitate the MBP domain. On the contrary, we have designed a different expression and purification protocol (Figure S1, Supporting Information) that uses IMAC affinity chromatography (based on the presence of a poly-His tag), allowing purification of the fusion protein while maintaining the binding pockets of the mSA2 and MBP domains unoccupied.

Finally, to build the modular system, a coil-coiled dimerization domain has been added. This domain is represented by an E_3_ coil motif bound to the C-terminal domain of the Gal-3(111), and a K_3_ coil domain fused to the N-terminal domain of the mSA2-MBP fusion protein (Figure 1C).

We have analyzed the binding capacity of the protein exploiting the different electrophoretic mobility of the ligands and the complex ligand-protein, as well as the intrinsic fluorescence of the ligands. When the capability of the fusion protein to bind fluorescent maltosylated or biotinylated ligands is assayed *in vitro* by gel electrophoresis, the binding of either ligand or both combined can be readily detected (Figure 4), indicating that after purification, the fusion protein retains both binding sites and constitutes a useful engineered protein for the delivery of cargos.

**Figure 4:**
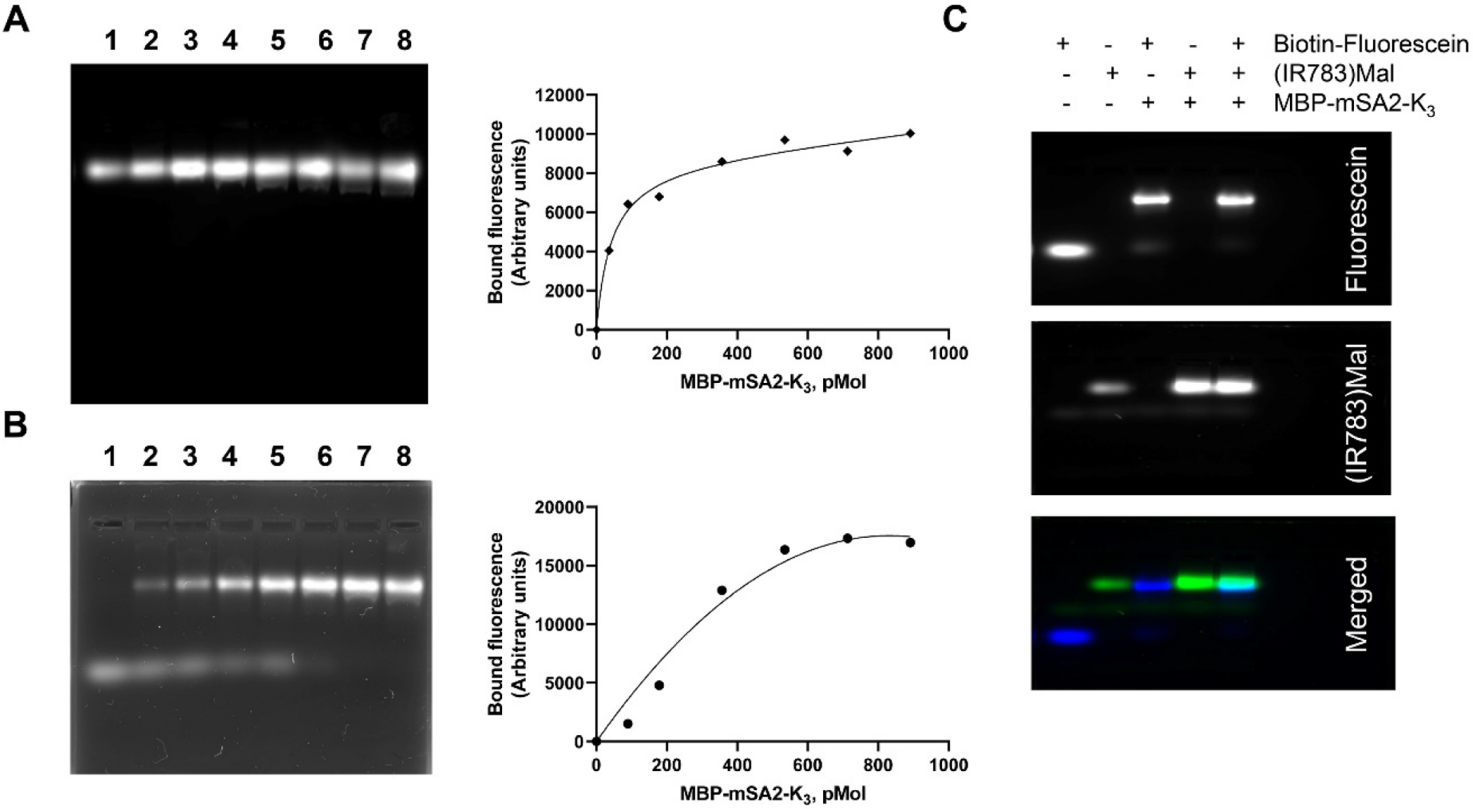
Binding of ligands to MBP-mSA2-K_3_. Either a fixed amount (35 pmol) of biotin-fluorescein (A) or MBP-NIR (B) has been preincubated for 30 min in the presence of increasing concentrations of MBP-mSA2-K_3_ protein (ranging from 0 to 900 pmol) and then analyzed by agarose electrophoresis. The binding of the ligands to the protein has been detected by changes in electrophoretic mobility (biotin-fluorescein) or by increased fluorescence intensity upon binding to the protein (maltose-NIR). (C) Biotin-fluorescein, maltose-NIR, or both have been incubated in the presence or absence of MBP-mSA2-K_3_ as indicated, and the binding of the ligands has been visualized after agarose electrophoresis at two emission wavelengths.

Next, we have assayed the ability to target the moiety to bind to the complex. As indicated, we have generated a fusion protein, termed Gal-3(111)-E_3_, that incorporates the E_3_ dimerization motif at the C termini. For the binding assay, we used agarose gel electrophoresis, and changes in their electrophoretic mobility detected the binding of the two proteins. While the Gal-3(111)-E_3_ protein does not migrate during the electrophoresis, the MBP-mSA2-K_3_ protein migrates toward the cathode, and the complex [Gal-3(111)-E_3_-MBP-mSA2-K_3_] has an intermediate mobility (Figure 5A). When increasing amounts of Gal-3(111)-E_3_ are added to a fixed amount of MBP-mSA2-K_3_ and electrophoresed, the changes in mobility reflect the formation of the [Gal-3(111)-E_3_-MBP-mSA2-K_3_] complex (Figure 5B).

**Figure 5:**
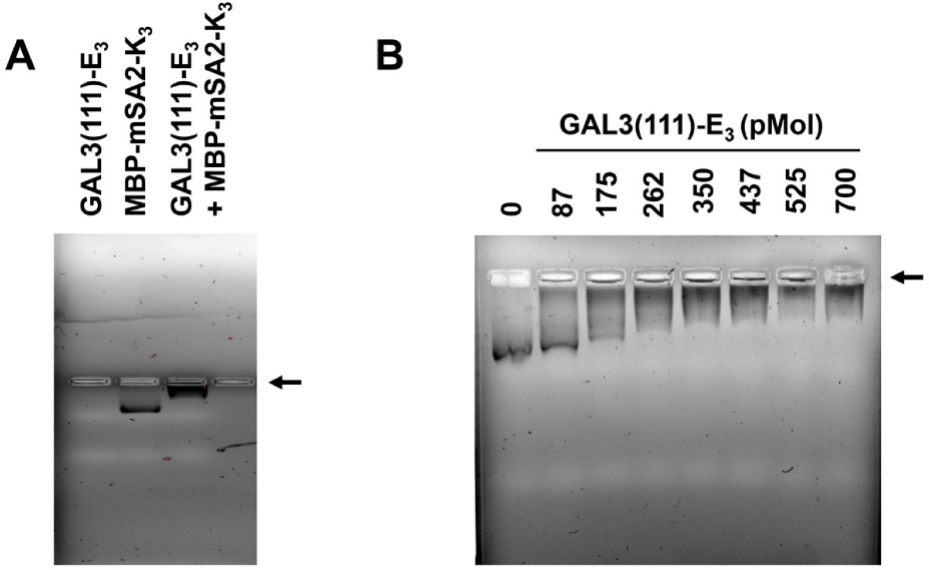
Binding of Gal-3(111)-E_3_ and MBP-mSA2-K_3_. (A) Equimolecular amounts of Gal-3(111)-E_3_ and MBP-mSA2-K_3_ (700 pmol) were incubated at room temperature for 10 min and then electrophoresed in TAE 0.8% agarose containing 5 μL/mL trichloroethanol. After electrophoresis, the gel was developed as described in the Materials and Methods section. (B) A fixed amount of Gal-3(111)-E_3_ (700 pmol) was incubated in the presence of increasing amounts of MBP-mSA2-K_3_ (0–700 pmol) for 10 min at room temperature and then electrophoresed in TAE agarose (0.8%) containing trichloroethanol (5 μL/mL). After electrophoresis, the gel was developed as described in the Materials and Methods section. Protein interactions were detected by changes in electrophoretic mobility. The arrows mark the electrophoretic mobility of the Gal-3(111)-E_3_ plus MBP-mSA2-K_3_ complex.

Once we have confirmed that the coil-coiled motif promotes the interaction between the Gal-3(111)-E_3_ directing molecule and the MBP-mSA2-K_3_ carrier protein, we have assayed the feasibility of delivering biotinylated cargos specifically to cells expressing MUC1. For that, cells that either express MUC1 (T47D and HeLa) or do not (HepG2) were used. Biotin-ATTO647 was selected as a biotinylated cargo to monitor cell uptake by fluorescence analysis and confocal microscopy (Figure 6).

**Figure 6:**
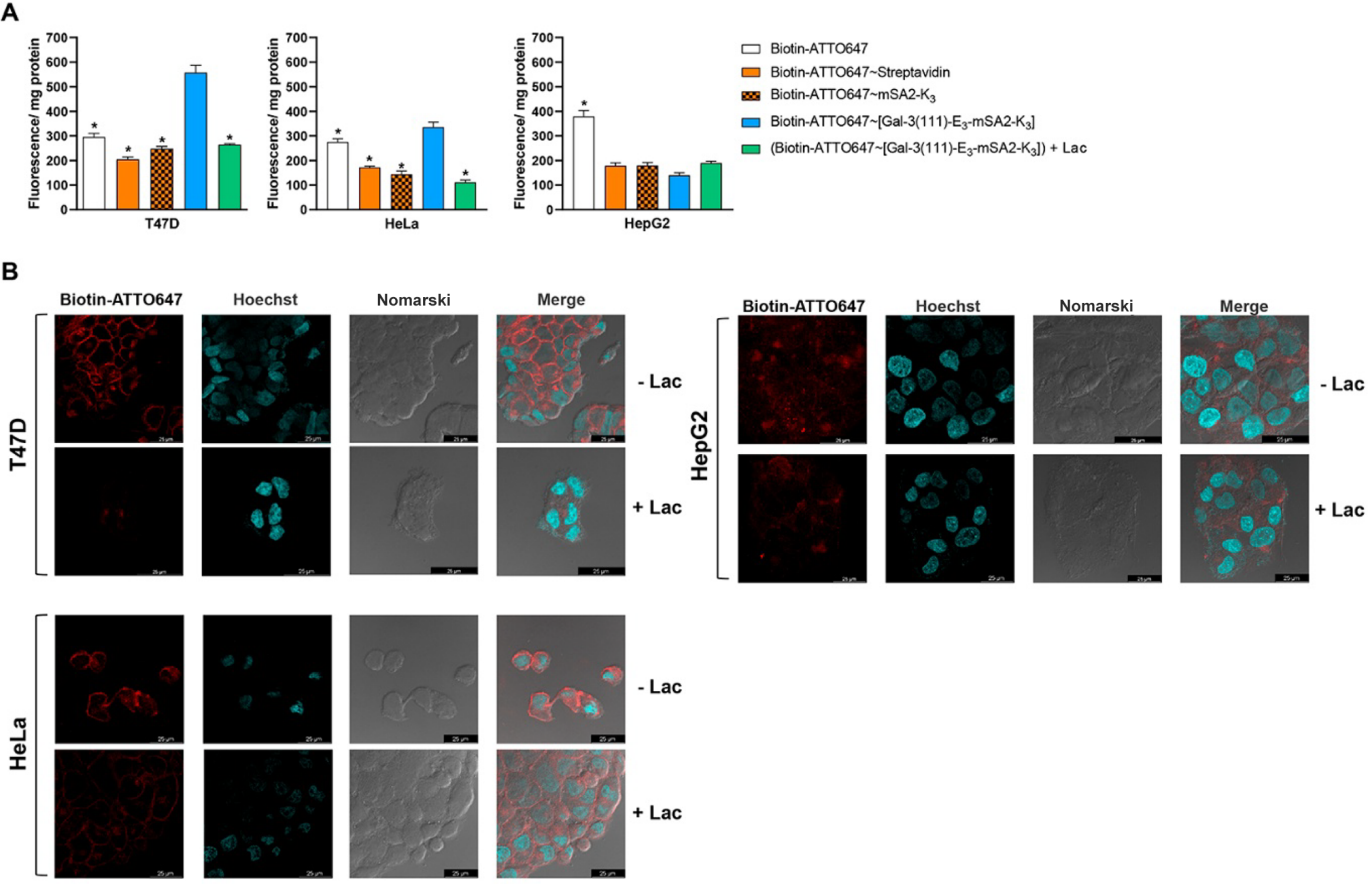
Targeting the capacity of biotin-ATTO647 bound to different ligands in cells expressing or not expressing the MUC1 receptor. (A) Uptake of biotin-ATTO647 bound to various proteins (10 μM) in MUC1-positive cells (T47D and HeLa) and negative cells (HepG2). The error bars represent SEM of six replicates. *P < 0.05 vs Biotin-ATTO647∼[Gal-3(111)-E_3_-MBP-mSA2-K_3_] treated cells. (B) Confocal images of T47D, HeLa and HepG2 cells incubated with Biotin-ATTO647∼[Gal-3(111)-E_3_-MBP-mSA2-K_3_] in the absence or presence of lactose (10 mM).

In cells expressing MUC1, the incorporation of Biotin-ATTO647 into the [Gal-3(111)-E_3_-MBP-mSA2-K_3_] complex translates into a significant increase in fluorescence uptake compared with the incubation with the biotinylated ligand alone. Furthermore, as negative controls, incubation with Biotin-ATTO647 bound to streptavidin or MBP-mSA2-K_3_, lacking the director moiety, decreased uptake. Also, preincubation with lactose of the Biotin-ATTO647∼[Gal-3(111)-E_3_-MBP-mSA2-K_3_] complex blocked the uptake. Finally, when HepG2 cells that do not express MUC1 were incubated with Biotin-ATTO647∼[Gal-3(111)-E_3_-MBP-mSA2-K_3_], a very low uptake was detected. Taken together, these results support the idea that [Gal-3(111)-E_3_-MBP-mSA2-K_3_] complex can transport biotinylated cargos specifically to cells that selectively express galactosylated ligands at the plasma membrane.

Once probed the capability of the [Gal-3(111)-E_3_-MBP-mSA2-K_3_] complex to deliver specifically fluorescent biotinylated cargos, we have exploited the incorporation of biotinyl groups into known drugs to enhance their specificity. As a model drug, we have selected doxorubicin (DOX). DOX is a well-known anthracycline with broad antitumor activity.^34^ Albeit largely used, it shows cumulative and dose-dependent cardiotoxicity, limiting its potential use.^35^ Several efforts have been made to increase the specificity of DOX toward cancer cells, for example, encapsulating it in cyclodextrins linked to targeting nanoparticles.^36^

We have modified the structure of DOX to introduce a biotin moiety (Figure 1D, Figure S2, Supporting Information) that could be specifically transported by the [Gal-3(111)-E_3_-MBP-mSA2-K_3_] complex. In this molecule, termed biotin-doxorubicin (b-DOX), the amino group of the DOX has been modified to introduce a spacer and a biotinyl group. Modifications of this amino group have been previously described to generate prodrugs that can be active in the cells.^37^ In addition, the linker between the DOX and the biotinyl group contains a sulfonate group that can be cleaved under reducing conditions, acting as a drug-release trigger,^38^ and freeing the DOX moiety. The decoupling of sulfonates with glutathione (GSH) under conditions compatible with those of living systems has been described,^39^ and therefore allows for the exploitation of this in some cancer cells, including HeLa cells, where the levels of reducing compounds, such as reduced glutathione, are elevated, preferentially facilitating the active drug release into these cells.^40^

First, we have compared the uptake of DOX and b-DOX in the HeLa and HepG2 cell lines, which serve as models of cells that overexpress or not MUC1 at the cell surface (Figure 7). Although HeLa and other cancer cells overexpress a cell surface receptor for biotinylated compounds, allowing it internalization,^41^ the presence of the biotin moiety in the b-DOX significantly reduces the drug uptake in both cell lines.

**Figure 7:**
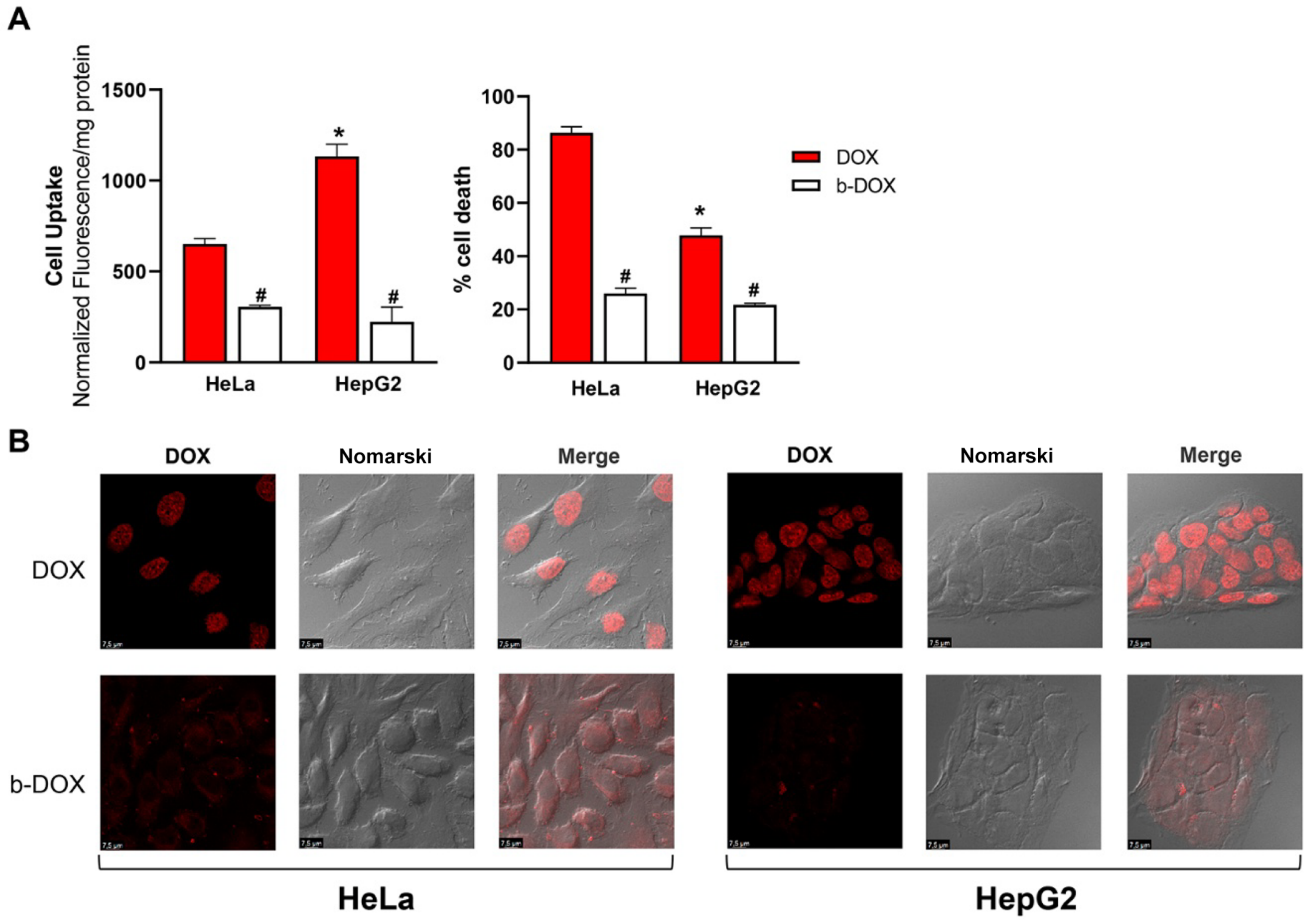
Effects of doxorubicin (DOX) and biotin-DOX (b-DOX) on cell uptake and viability in cells expressing or not the MUC1 receptor. (**A**) HeLa (MUC1+) and HepG2 (MUC1−) cells were incubated with DOX or b-DOX (27 μM) to measure uptake (2 h) and cell death (48 h). Results were normalized to 0% for control cells. The error bars represent SEM of six replicates. *p < 0.05 vs HeLa cells incubated with DOX. #*p* < 0.05 vs DOX incubated cells. Confocal photos (**B**) were taken in HeLa or HepG2 cells incubated with DOX or b-DOX (27 μM) for 2 h. Results are mean ± SEM (*n* = 6).

The decreased with b-DOX uptake parallels the lower cell death in both cell lines when compared with the DOX treatment. Interestingly, when cell uptake is analyzed by confocal microscopy (Figure 7B), at 2 h of incubation, while DOX is mainly located in the cell nucleus, b-DOX is located outside the nucleus, probably associated with endosomes, as a reflection of the biotin receptor-mediated uptake compared to the passive diffusion of DOX. Therefore, although the inclusion of biotin in the DOX structure results in increased specificity to cancer cell lines overexpressing biotin receptors, the decrease in toxicity in these cells could constitute a major drawback for its effectiveness as an antitumoral agent.

We have analyzed cell uptake and cell-induced toxicity of the b-DOX complexed with [Gal-3(111)-E_3_-MBP-mSA2-K_3_] in the MUC1-expressing HeLa cell line, and as a negative control the HepG2 cells (Figure 8).

**Figure 8:**
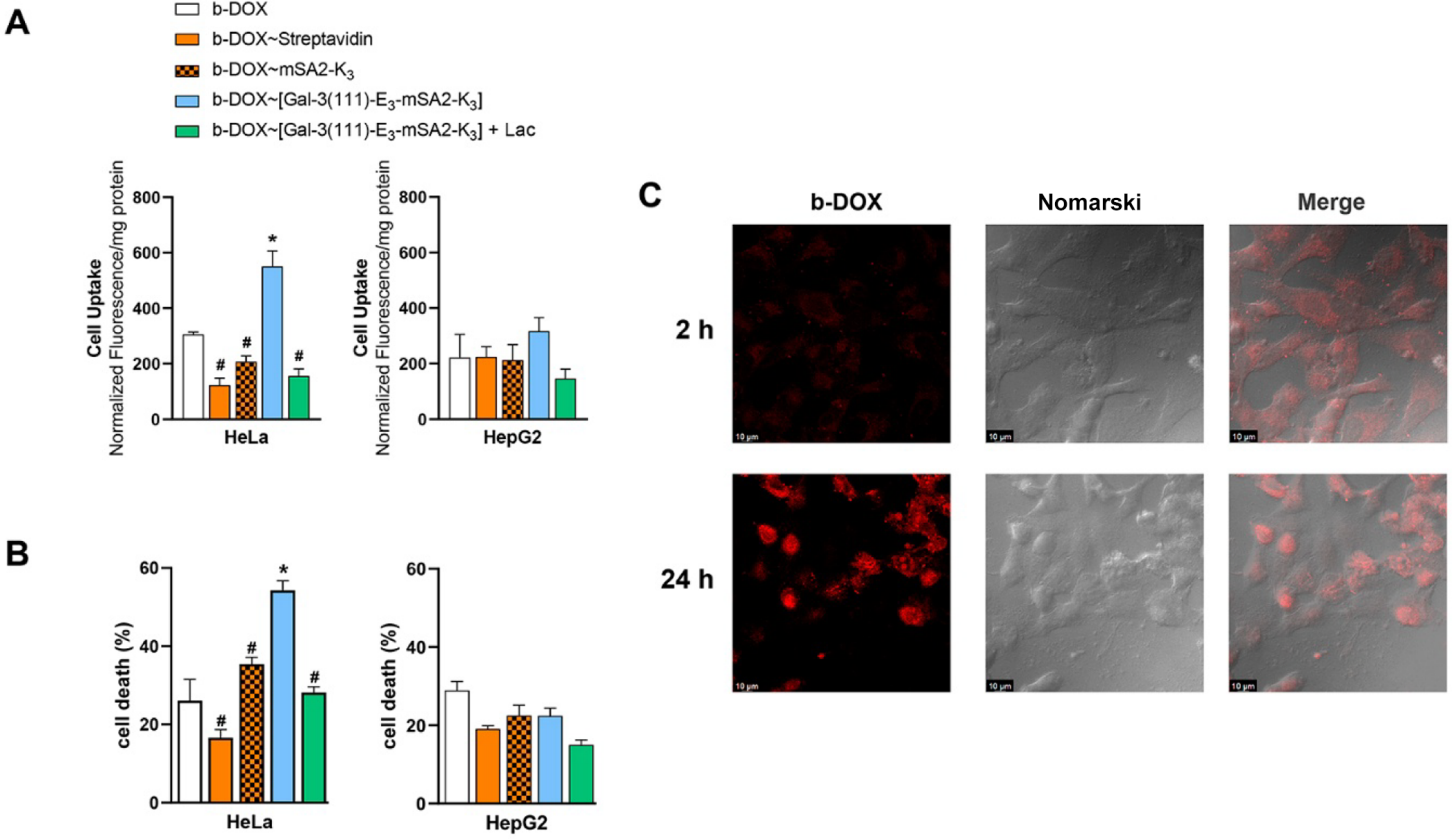
Effects of biotin-DOX (b-DOX) combined with various proteins on cell uptake and viability in cells expressing or not the MUC1 receptor. Cells were incubated with b-DOX, which binds to various proteins (10 μM) to measure uptake (2 h) (**A**) and cell death (48 h) (**B**). The error bars represent SEM of six replicates. *P < 0.05 vs b-DOX treated cells and #*p* < 0.05 vs the b-DOX complexed with [Gal-3(111)-E_3_-MBP-mSA2-K_3_]. (**C**) Confocal images of cells incubated with b-DOX-[Gal-3(111)-E_3_-MBP-mSA2-K_3_] for 2 h (upper panel) and 24 h (lower panel).

Regarding uptake experiments, the results obtained in HeLa cells using b-DOX as a cargo molecule are like those obtained with Biotin-ATTO647 (Figure 7). While the uptake of b-DOX is lower than that of the unmodified DOX (Figure 7), complexation with [Gal-3(111)-E_3_-MBP-mSA2-K_3_] significantly enhances the uptake. On the contrary, complexation with streptavidin or only the MBP-mSA2-K_3_ protein substantially decreases the uptake. Also, preincubation with lactose significantly blocks the uptake mediated by the targeting complex. On the contrary, in HepG2 cells, which lack MUC1 expression at the plasma membrane, the uptake of b-DOX is not increased upon incubation with any protein, remaining only at the values obtained by biotin receptor-mediated internalization.

In parallel with b-DOX uptake, cell toxicity induced by incubation with the b-DOX complexed with [Gal-3(111)-E_3_-MBP-mSA2-K_3_] was significantly higher in HeLa cells than in cells incubated with b-DOX alone, with cell death values close to those obtained upon incubation with unmodified DOX. As expected, b-DOX-induced death was similar for all conditions tested in the HepG2 cells.

A time course of intracellular trafficking of b-DOX∼[Gal-3(111)-E_3_-MBP-mSA2-K_3_] in HeLa cells has been performed using confocal microscopy (Figure 8C). After 2 h of incubation, the fluorescence shows mainly cytosolic distribution, like that of Biotin-ATTO647∼[Gal-3(111)-E_3_-MBP-mSA2-K_3_] (Figure 6B). After 24 h, the fluorescence predominantly translocates to the cell nucleus, likely due to sulfonate group hydrolysis, releasing DOX, which can then migrate to the nucleus. This is significant because DOX’s cytotoxic effect is partly exerted in the nucleus by inhibiting topoisomerase II activity.^42^

The main point of the proposed strategy is its modularity. To demonstrate this point, the targeting moiety based on Gal-3(111) has been replaced with an anti-HER2 single-chain antibody (HER2 ScFv) fused to the E_3_ coil motif. This construct is based on a previously used contruct^7^ where the HER2 ScFv fused to the MBP is used to deliver maltosylated ligands to cells expressing HER2. In Figure S4, Supporting Information, the capability to form the complex [HER2 ScFv-E_3_-MBP-mSA2-K_3_] as well as the delivery of Biotin-ATTO647∼[HER2 ScFv-E_3_-MBP-mSA2-K_3_] to cells expressing (SKBR3 cells) or not (MDA-MB 231) the HER2 receptors. Our data confirm that the new complex can specifically target the HER2 surface receptor.

Finally, to evaluate the potential of the targeting molecule as a site-directed drug-delivery system, an *in vivo* analysis was carried out using immunosuppressed NSG mice bearing HeLa cell xenografts (Figure 9). *In vivo* imaging systems are not optimal for detecting DOX due to the relatively low wavelength of its fluorescence emission; therefore, biotin-ATTO740 was used as a model for drug delivery instead of b-DOX.

**Figure 9:**
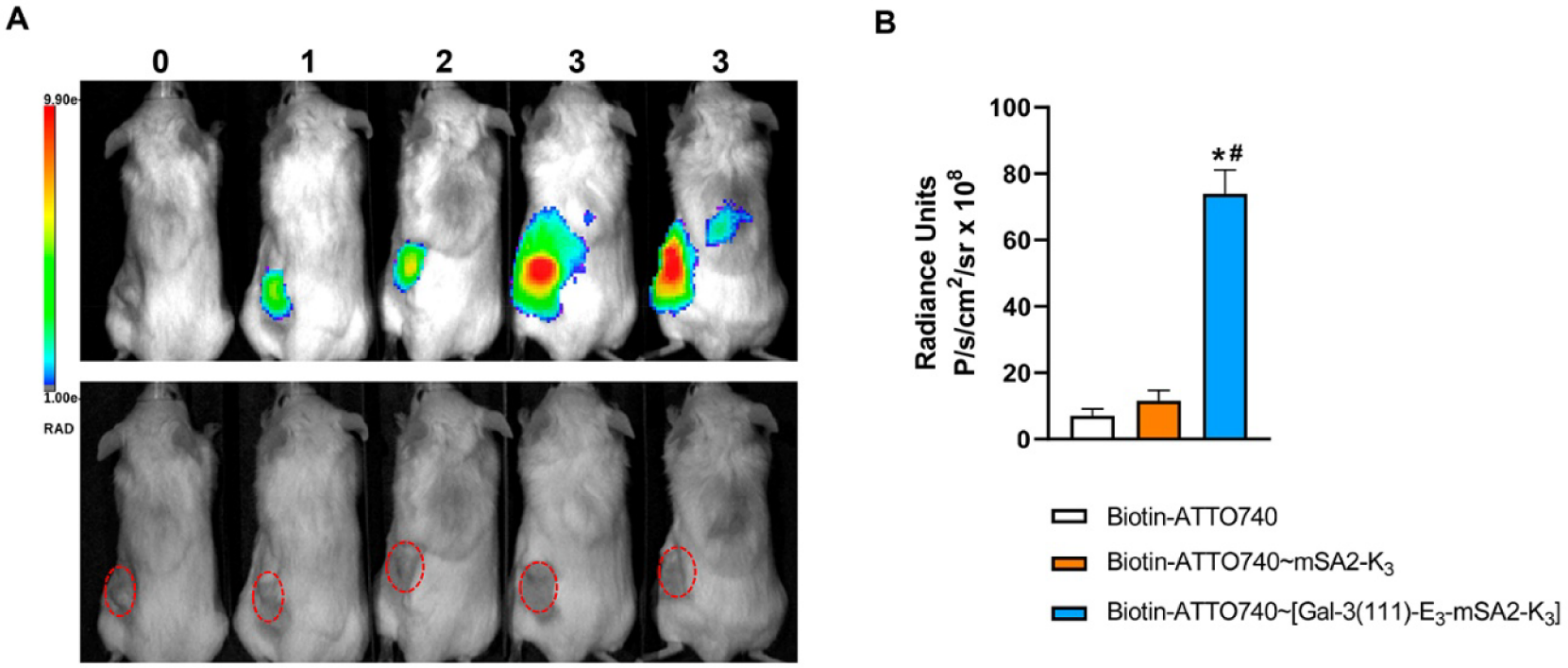
Biotin-ATTO740∼[Gal-3­(111)-E_3_-mSA2-K_3_] targeted delivery to mice bearing HeLa cells xenografts. (**A**) *In vivo* delivery for NSG mice bearing HeLa tumor-xenografts. Images were taken 24 h after the compound’s intravenous injection in the tail vein. 0: Control; 1: Biotin-ATTO740; 2: Biotin-ATTO740∼mSA2-K_3_; 3: Biotin-ATTO740∼[Gal-3(111)-E_3_-mSA2-K_3_]. The size of the xenografts is indicated by a red dotted line in the lower panel. (**B**) The average radiance of the xenografts from the mice injected with the compounds is plotted. The error bars represent SEM of four replicates. *p < 0.05 vs Biotin-ATTO740 xenografts. #*p* < 0.05 vs Biotin-ATTO740∼mSA2-K_3_ xenografts.

In animals, the analysis of the distribution of the fluorescence after 24 h from the injection in the tail vein revealed that Biotin-ATTO740∼[Gal-3(111)-E_3_-MBP-mSA2-K_3_] was visible at the xenograft, and quantification of the fluorescence yielded an 8-fold higher fluorescence than xenografts from animals injected with Biotin-ATTO740 or Biotin-ATTO740-MBP-mSA2-K_3_.

## 4 Conclusions

The search for new cancer markers to enable specific, personalized therapy is needed. Although numerous surface proteins can be targeted in cancer cells, the genetic variability of tumors means that many are considered negative for a specific molecular target. Therefore, broadening the panoply of cell surface target molecules in cancer is required to avoid conventional chemotherapy. This article highlights the feasibility of using human Gal-3 as a new targeting moiety for glycosylated surface proteins in cancer cells. Gal-3 is a human lectin of reduced size, and even more so its truncated version Gal-3(111), which contains only the carbohydrate recognition domain, is well suited as a targeting moiety, probably avoiding the stimulation of proliferative pathways. Another main point in the article is the development of a versatile, modular, and interchangeable theranostic agent. For that, a carrier fusion protein that can bind two cargo molecules is presented. For visualization, a maltosylated near-infrared fluorophore has been selected, while for the therapeutic agent, a biotinylated DOX has been used. By combining the carrier fusion protein with the director protein via a coil-coiled motif, it is possible to interchange the director protein without any additional modification easily. The targeting specificity has been demonstrated in cell culture and xenografts. Further experiments are needed to prove the efficacy of this therapeutic approach in animals.

## Supplementary Material



## Notes

Oligonucleotides used; sequences of plasmids used; expression and purification of recombinant proteins used in this article; synthesis and properties of biotin-doxorubicin; silencing of the expression of MUC1 in HeLa cells using CRISPR-Cas9; targeting of HER2 receptor-expressing cells­(PDF)

## Abbreviations

b-DOX: Biotin-doxorubicin
DMEM: Dulbecco’s modified Eagle’s medium
DOX: Doxorubicin
E_3_-K_3_: Coil-coiled protein domain
FBS: Fetal bovine serum
FITC: Fluorescein isothiocyanate isomer I
Gal-3: Galectin-3
gRNA: Guide RNA;
GSH: Glutathione
IMAC: Immobilized metal affinity chromatography
LAMP-1: Lysosomal-associated membrane protein 1
MBP: Maltose-binding protein
mSA2: Monomeric modified streptavidin
MUC1: Mucin 1
NSG mice: NOD scid gamma mice
PAM: Protospacer adjacent motif
PBS: Phosphate-buffered saline
SDS–PAGE: Sodium dodecyl sulfate polyacrylamide gel electrophoresis
TAE buffer: Tris acetate-EDTA buffer
