# Supplementary material for: Engineering Coil-Coiled Domains for the Design of Modular Theranostic Agents: Galectin‑3 as a Targeting Moiety
Source: Biomacromolecules. 2026 Jun 27;27(7):4597–608. doi: 10.1021/acs.biomac.6c00502 (PMC13370767; doi:10.1021/acs.biomac.6c00502)

ASSOCIATED CONTENT

Supporting Information

ENGINEERING COIL-COILED DOMAINS FOR THE DESIGN OF MODULAR THERANOSTIC AGENTS: GALECTIN-3 AS TARGETING MOIETY

*CHIARA BURGIO, MARIA D. GIRON, MARIANO ORTEGA-MUÑOZ, DANIEL LUCENA, AND RAFAEL SALTO**

Table of contents

1. **Table S1.** *Oligonucleotides used in this article.*
2. *Sequences of plasmids used in this article.*
3. **Fig. S1.** *Expression and purification of the recombinant protein used in this article.*
4. **Fig. S2.** *Synthesis of biotin-doxorubicin*.
5. **Figure S3.** *Silencing of the expression of MUC1 in HeLa cells using CRISPR Cas9*
6. **Figure S4.** *Targeting the HER2 receptor in SKBR3 cells using a Coil-coiled system*

1. **Table S1.** *Oligonucleotides used in this article.*

Name	Sequence	Restrict. site	Purpose
**Gal3-f**	5´-ggatccATGGCCCAGGTGCAGCTGGTG-3´	*BamH*I	Gal3 Cloning in pMAL-TEV-His
**Gal3(111)-f**	5´-ggatccGCTGGGCCACTGATT-3´	*BamH*I	Gal3(111) Cloning in pMAL-TEV-His
**Gal3-r**	5´-gtcgacTATCATGGTATATGAAGC-3´	*Sal*I	Gal3 and Gal3(111) Cloning in pMAL-TEV-His
**Ser-link-f**	5´-tcgacGGCGGAGGAGGATCTGGCGGAGGAGGATCT ctgca-3`	*Sal*I and *Pst*I	Linker between Gal3 and coil
**Ser-link-r**	5´-gAGATCCTCCTCCGCCAGATCCTCCTCCGCCg-3´	*Sal*I and *Pst*I	Linker between Gal3 and coil
**E3-f**	5´-gGCCATGGAAATCGCCGCTCTAGAAAAAGAGATC GCTGCTCTGGAGAAGGAGATTGCCGCCCTTGAGAAG GGCGGCgc-3´	*Nco*I *and Not*I	Linker that codes for E3 coil
**E3-r**	5´-ggccgcGCCGCCCTTCTCAAGGGCGGCAATCTCCTTC TCCAAGCAGCGATCTCTTTTTCTAGAGCGGCGATTTC CATGGCctgca-3´	*Nco*I *and Not*I	Linker that codes for E3 coil
**mSA2-f**	5´-ggatccGCGGAAGCGGGTATCAC-3´	*BamH*I	mSA2 Cloning in pMAL-TEV-His
**mSA2-r**	5´-ccatggAACTGCTGTTTCCAGACGCCGCAGACGGTT TAACTTTGGTGAAGG-3´	*Nco*I	mSA2 Cloning in pMAL-TEV-His
**K3-f**	5´-catggCCATGAAGATCGCCGCCCTGAAGGAGAAGAT CGCCGCCCTGAAGGAGAAGATCGCCGCCCTTAAGGA GGGCGgcGC-3´	*Pst*I *and Not*I	Linker that codes for K3 coil
**K3-r**	5´-gGCCGCgcCGCCCTCCTTAAGGGCGGCGATCTTCTC CTTCAGGGCGGCGATCTTCTCCTTCAGGGCGGCGATC TTCATGGc-3´	*Pst*I *and Not*I	Linker that codes for K3 coil
**gMUC1-f**	5´-CACC-G-AAGAAAGGAGACTGGGTGCC-3´		Silencing human muc1 using CRISPR Cas9
**gMUC1-r**	5´-AAAC-GGCACCCAGTCTCCTTTCTT-3´		Silencing human muc1 using CRISPR Cas9

2. Sequences of plasmids used in this article.

pMAL-TEV-Gal3-His

5´-ccgacaccatcgaatggtgcaaaacctttcgcggtatggcatgatagcgcccggaagagag
tcaattcagggtggtgaatgtgaaaccagtaacgttatacgatgtcgcagagtatgccggtgtc
tcttatcagaccgtttcccgcgtggtgaaccaggccagccacgtttctgcgaaaacgcgggaaa
aagtggaagcggcgatggcggagctgaattacattcccaaccgcgtggcacaacaactggcggg
caaacagtcgttgctgattggcgttgccacctccagtctggccctgcacgcgccgtcgcaaatt
gtcgcggcgattaaatctcgcgccgatcaactgggtgccagcgtggtggtgtcgatggtagaac
gaagcggcgtcgaagcctgtaaagcggcggtgcacaatcttctcgcgcaacgcgtcagtgggct
gatcattaactatccgctggatgaccaggatgccattgctgtggaagctgcctgcactaatgtt
ccggcgttatttcttgatgtctctgaccagacacccatcaacagtattattttctcccatgaag
acggtacgcgactgggcgtggagcatctggtcgcattgggtcaccagcaaatcgcgctgttagc
gggcccattaagttctgtctcggcgcgtctgcgtctggctggctggcataaatatctcactcgc
aatcaaattcagccgatagcggaacgggaaggcgactggagtgccatgtccggttttcaacaaa
ccatgcaaatgctgaatgagggcatcgttcccactgcgatgctggttgccaacgatcagatggc
gctgggcgcaatgcgcgccattaccgagtccgggctgcgcgttggtgcggatatctcggtagtg
ggatacgacgataccgaagacagctcatgttatatcccgccgttaaccaccatcaaacaggatt
ttcgcctgctggggcaaaccagcgtggaccgcttgctgcaactctctcagggccaggcggtgaa
gggcaatcagctgttgcccgtctcactggtgaaaagaaaaaccaccctggcgcccaatacgcaa
accgcctctccccgcgcgttggccgattcattaatgcagctggcacgacaggtttcccgactgg
aaagcgggcagtgagcgcaacgcaattaatgtaagttagctcactcattaggcacaattctcat
gtttgacagcttatcatcgactgcacggtgcaccaatgcttctggcgtcaggcagccatcggaa
gctgtggtatggctgtgcaggtcgtaaatcactgcataattcgtgtcgctcaaggcgcactccc
gttctggataatgttttttgcgccgacatcataacggttctggcaaatattctgaaatgagctg
ttgacaattaatcatcggctcgtataatgtgtggaattgtgagcggataacaatttcacacagg
aaacagccagtccgtttaggtgttttcacgagcacttcaccaacaaggaccatagcatatgaaa
atcgaagaaggtaaactggtaatctggattaacggcgataaaggctataacggtctcgctgaag
tcggtaagaaattcgagaaagataccggaattaaagtcaccgttgagcatccggataaactgga
agagaaattcccacaggttgcggcaactggcgatggccctgacattatcttctgggcacacgac
cgctttggtggctacgctcaatctggcctgttggctgaaatcaccccggacaaagcgttccagg
acaagctgtatccgtttacctgggatgccgtacgttacaacggcaagctgattgcttacccgat
cgctgttgaagcgttatcgctgatttataacaaagatctgctgccgaacccgccaaaaacctgg
gaagagatcccggcgctggataaagaactgaaagcgaaaggtaagagcgcgctgatgttcaacc
tgcaagaaccgtacttcacctggccgctgattgctgctgacggggttatgcgttcaagtatga
aaacggcaagtacgacattaaagacgtgggcgtggataacgctggcgcgaaagcgggtctgacc
ttcctggttgacctgattaaaaacaaacacatgaatgcagacaccgattactccatcgcagaag
ctgcctttaataaaggcgaaacagcgatgaccatcaacggcccgtgggcatggtccaacatcga
caccagcaaagtgaattatggtgtaacggtactgccgaccttcaagggtcaaccatccaaaccg
ttcgttggcgtgctgagcgcaggtattaacgccgccagtccgaacaaagagctggcaaaagagt
tcctcgaaaactatctgctgactgatgaaggtctggaagcggttaataaagacaaaccgctggg
tgccgtagcgctgaagtcttacgaggaagagttggcgaaagatccacgtattgccgccactatg
gaaaacgcccagaaaggtgaaatcatgccgaacatcccgcagatgtccgctttctggtatgccg
tgcgtactgcggtgatcaacgccgccagcggtcgtcagactgtcgatgaagccctgaaagacgc

gcagactaattcgagctcgaacaacaacaacaataacaataacaacaacctcgggagctcggag
aatctttattttcagggcggatccatggcagacaatttttcgctccatgatgcgttatctgggt
ctggaaacccaaaccctcaaggatggcctggcgcatggggggaaccagcctgctggggcagggg
ctacccaggggcttcctatcctggggcctaccccgggcaggcacccccaggggcttatcctgga
caggcacctccaggcgcctaccctggagctcctggagcttatcccggagcacctgcacctggag
tctacccagggccacccagcggccctggggcctacccatcttctggacagccaagtgccaccgg
tgcctaccctgccactggcccctatggcgcccctgctgggccactgattgtgccttataacctg
cctttgcctgggggagtggtgcctcgcatgctgataacaattctgggcacggtgaagcccaatg
caaacagaattgctttagatttccaaagagggaatgatgttgccttccactttaacccacgctt
caatgagaacaacaggagagtcattgtttgcaatacaaagctggataataactggggaagggaa
gaaagacagtcggttttcccatttgaaagtgggaaaccattcaaaatacaagtactggttgaac
ctgaccacttcaaggttgcagtgaatgatgctcacttgttgcagtacaatcatcgggttaaaaa
actcaatgaaatcagcaaactgggaatttctggtgacatagacctcaccagtgcttcatatacc
atgatagtcgacctgcaggcggccgccggcggaggaggatctcatcatcaccaccaccattaag
cttggactggccgtcgttttacaacgtcgtgactgggaaaaccctggcgttacccaacttaatc
gccttgcagcacatccccctttcgccagctggcgtaatagcgaagaggcccgcaccgatcgccc
ttcccaacagttgcgcagcctgaatggcgaatggcagcttggctgttttggcggatgagataag
attttcagcctgatacagattaaatcagaacgcagaagcggtctgataaaacagaatttgcctg
gcggcagtagcgcggtggtcccacctgaccccatgccgaactcagaagtgaaacgccgtagcgc
cgatggtagtgtggggtctccccatgcgagagtagggaactgccaggcatcaaataaaacgaaa
ggctcagtcgaaagactgggcctttcgttttatctgttgtttgtcggtgaacgctctcctgagt
aggacaaatccgccgggagcggatttgaacgttgcgaagcaacggcccggagggtggcgggcag
gacgcccgccataaactgccaggcatcaaattaagcagaaggccatcctgacggatggcctttt
tgcgtttctacaaactctttttgtttatttttctaaatacattcaaatatgtatccgctcatga
gacaataaccctgataaatgcttcaataatattgaaaaaggaagagtatgagtattcaacattt
ccgtgtcgcccttattcccttttttgcggcattttgccttcctgtttttgctcacccagaaacg
ctggtgaaagtaaaagatgctgaagatcagttgggtgcacgagtgggttacatcgaactggatc
tcaacagcggtaagatccttgagagttttcgccccgaagaacgttctccaatgatgagcacttt
taaagttctgctatgtggcgcggtattatcccgtgttgacgccgggcaagagcaactcggtcgc
cgcatacactattctcagaatgacttggttgagtactcaccagtcacagaaaagcatcttacgg
atggcatgacagtaagagaattatgcagtgctgccataaccatgagtgataacactgcggccaa
cttacttctgacaacgatcggaggaccgaaggagctaaccgcttttttgcacaacatgggggat
catgtaactcgccttgatcgttgggaaccggagctgaatgaagccataccaaacgacgagcgtg
acaccacgatgcctgtagcaatggcaacaacgttgcgcaaactattaactggcgaactacttac
tctagcttcccggcaacaattaatagactggatggaggcggataaagttgcaggaccacttctg
cgctcggcccttccggctggctggtttattgctgataaatctggagccggtgagcgtgggtctc
gcggtatcattgcagcactggggccagatggtaagccctcccgtatcgtagttatctacacgac
ggggagtcaggcaactatggatgaacgaaatagacagatcgctgagataggtgcctcactgatt
aagcattggtaactgtcagaccaagtttactcatatatactttagattgatttaccccggttga
taatcagaaaagccccaaaaacaggaagattgtataagcaaatatttaaattgtaaacgttaat
attttgttaaaattcgcgttaaatttttgttaaatcagctcattttttaaccaataggccgaaa
tcggcaaaatcccttataaatcaaaagaatagaccgagatagggttgagtgttgttccagtttg
gaacaagagtccactattaaagaacgtggactccaacgtcaaagggcgaaaaaccgtctatcag
ggcgatggcccactacgtgaaccatcacccaaatcaagttttttggggtcgaggtgccgtaaag
cactaaatcggaaccctaaagggagcccccgatttagagcttgacggggaaagccggcgaacgt
ggcgagaaaggaagggaagaaagcgaaaggagcgggcgctagggcgctggcaagtgtagcggtc
acgctgcgcgtaaccaccacacccgccgcgcttaatgcgccgctacagggcgcgtaaaaggatc

taggtgaagatcctttttgataatctcatgaccaaaatcccttaacgtgagttttcgttccact
gagcgtcagaccccgtagaaaagatcaaaggatcttcttgagatcctttttttctgcgcgtaat
ctgctgcttgcaaacaaaaaaaccaccgctaccagcggtggtttgtttgccggatcaagagcta
ccaactctttttccgaaggtaactggcttcagcagagcgcagataccaaatactgtccttctag
tgtagccgtagttaggccaccacttcaagaactctgtagcaccgcctacatacctcgctctgct
aatcctgttaccagtggctgctgccagtggcgataagtcgtgtcttaccgggttggactcaaga
cgatagttaccggataaggcgcagcggtcgggctgaacggggggttcgtgcacacagcccagct
tggagcgaacgacctacaccgaactgagatacctacagcgtgagctatgagaaagcgccacgct
tcccgaagggagaaaggcggacaggtatccggtaagcggcagggtcggaacaggagagcgcacg
agggagcttccagggggaaacgcctggtatctttatagtcctgtcgggtttcgccacctctgac
ttgagcgtcgatttttgtgatgctcgtcaggggggcggagcctatggaaaaacgccagcaacgc
ggcctttttacggttcctggccttttgctggccttttgctcacatgttctttcctgcgttatcc
cctgattctgtggataaccgtattaccgcctttgagtgagctgataccgctcgccgcagccgaa
cgaccgagcgcagcgagtcagtgagcgaggaagcggaagagcgcctgatgcggtattttctcct
tacgcatctgtgcggtatttcacaccgcatatatggtgcactctcagtacaatctgctctgatg
ccgcatagttaagccagtatacactccgctatcgctacgtgactgggtcatggctgcgccccga
cacccgccaacacccgctgacgcgccctgacgggcttgtctgctcccggcatccgcttacagac
aagctgtgaccgtctccgggagctgcatgtgtcagaggttttcaccgtcatcaccgaaacgcgc
gaggcagctgcggtaaagctcatcagcgtggtcgtgcagcgattcacagatgtctgcctgttca
tccgcgtccagctcgttgagtttctccagaagcgttaatgtctggcttctgataaagcgggcca
tgttaagggcggttttttcctgtttggtcactgatgcctccgtgtaagggggatttctgttcat
gggggtaatgataccgatgaaacgagagaggatgctcacgatacgggttactgatgatgaacat
gcccggttactggaacgttgtgagggtaaacaactggcggtatggatgcggcgggaccagagaa
aaatcactcagggtcaatgccagcgcttcgttaatacagatgtaggtgttccacagggtagcca
gcagcatcctgcgatgcagatccggaacataatggtgcagggcgctgacttccgcgtttccaga
ctttacgaaacacggaaaccgaagaccattcatgttgttgctcaggtcgcagacgttttgcagc
agcagtcgcttcacgttcgctcgcgtatcggtgattcattctgctaaccagtaaggcaaccccg
ccagcctagccgggtcctcaacgacaggagcacgatcatgcgcacccgtggccaggacccaacg
ctgcccgaaatt-3´

pMAL-TEV-Gal3(111)-His

5´-ccgacaccatcgaatggtgcaaaacctttcgcggtatggcatgatagcgcccggaagagag
tcaattcagggtggtgaatgtgaaaccagtaacgttatacgatgtcgcagagtatgccggtgtc
tcttatcagaccgtttcccgcgtggtgaaccaggccagccacgtttctgcgaaaacgcgggaaa
aagtggaagcggcgatggcggagctgaattacattcccaaccgcgtggcacaacaactggcggg
caaacagtcgttgctgattggcgttgccacctccagtctggccctgcacgcgccgtcgcaaatt
gtcgcggcgattaaatctcgcgccgatcaactgggtgccagcgtggtggtgtcgatggtagaac
gaagcggcgtcgaagcctgtaaagcggcggtgcacaatcttctcgcgcaacgcgtcagtgggct
gatcattaactatccgctggatgaccaggatgccattgctgtggaagctgcctgcactaatgtt
ccggcgttatttcttgatgtctctgaccagacacccatcaacagtattattttctcccatgaag
acggtacgcgactgggcgtggagcatctggtcgcattgggtcaccagcaaatcgcgctgttagc
gggcccattaagttctgtctcggcgcgtctgcgtctggctggctggcataaatatctcactcgc
aatcaaattcagccgatagcggaacgggaaggcgactggagtgccatgtccggttttcaacaaa
ccatgcaaatgctgaatgagggcatcgttcccactgcgatgctggttgccaacgatcagatggc
gctgggcgcaatgcgcgccattaccgagtccgggctgcgcgttggtgcggatatctcggtagtg

ggatacgacgataccgaagacagctcatgttatatcccgccgttaaccaccatcaaacaggatt
ttcgcctgctggggcaaaccagcgtggaccgcttgctgcaactctctcagggccaggcggtgaa
gggcaatcagctgttgcccgtctcactggtgaaaagaaaaaccaccctggcgcccaatacgcaa
accgcctctccccgcgcgttggccgattcattaatgcagctggcacgacaggtttcccgactgg
aaagcgggcagtgagcgcaacgcaattaatgtaagttagctcactcattaggcacaattctcat
gtttgacagcttatcatcgactgcacggtgcaccaatgcttctggcgtcaggcagccatcggaa
gctgtggtatggctgtgcaggtcgtaaatcactgcataattcgtgtcgctcaaggcgcactccc
gttctggataatgttttttgcgccgacatcataacggttctggcaaatattctgaaatgagctg
ttgacaattaatcatcggctcgtataatgtgtggaattgtgagcggataacaatttcacacagg
aaacagccagtccgtttaggtgttttcacgagcacttcaccaacaaggaccatagcatatgaaa
atcgaagaaggtaaactggtaatctggattaacggcgataaaggctataacggtctcgctgaag
tcggtaagaaattcgagaaagataccggaattaaagtcaccgttgagcatccggataaactgga
agagaaattcccacaggttgcggcaactggcgatggccctgacattatcttctgggcacacgac
cgctttggtggctacgctcaatctggcctgttggctgaaatcaccccggacaaagcgttccagg
acaagctgtatccgtttacctgggatgccgtacgttacaacggcaagctgattgcttacccgat
cgctgttgaagcgttatcgctgatttataacaaagatctgctgccgaacccgccaaaaacctgg
gaagagatcccggcgctggataaagaactgaaagcgaaaggtaagagcgcgctgatgttcaacc
tgcaagaaccgtacttcacctggccgctgattgctgctgacggggttatgcgttcaagtatga
aaacggcaagtacgacattaaagacgtgggcgtggataacgctggcgcgaaagcgggtctgacc
ttcctggttgacctgattaaaaacaaacacatgaatgcagacaccgattactccatcgcagaag
ctgcctttaataaaggcgaaacagcgatgaccatcaacggcccgtgggcatggtccaacatcga
caccagcaaagtgaattatggtgtaacggtactgccgaccttcaagggtcaaccatccaaaccg
ttcgttggcgtgctgagcgcaggtattaacgccgccagtccgaacaaagagctggcaaaagagt
tcctcgaaaactatctgctgactgatgaaggtctggaagcggttaataaagacaaaccgctggg
tgccgtagcgctgaagtcttacgaggaagagttggcgaaagatccacgtattgccgccactatg
gaaaacgcccagaaaggtgaaatcatgccgaacatcccgcagatgtccgctttctggtatgccg
tgcgtactgcggtgatcaacgccgccagcggtcgtcagactgtcgatgaagccctgaaagacgc
gcagactaattcgagctcgaacaacaacaacaataacaataacaacaacctcgggagctcggag
aatctttattttcagggcggatccgctgggccactgattgtgccttataacctgcctttgcctg
gggagtggtgcctcgcatgctgataacaattctgggcacggtgaagcccaatgcaaacagaat
tgctttagatttccaaagagggaatgatgttgccttccactttaacccacgcttcaatgagaac
aacaggagagtcattgtttgcaatacaaagctggataataactggggaagggaagaaagacagt
cggttttcccatttgaaagtgggaaaccattcaaaatacaagtactggttgaacctgaccactt
caaggttgcagtgaatgatgctcacttgttgcagtacaatcatcgggttaaaaaactcaatgaa
atcagcaaactgggaatttctggtgacatagacctcaccagtgcttcatataccatgatagtcg
acctgcaggcggccgccggcggaggaggatctcatcatcaccaccaccattaagcttggcactg
gccgtcgttttacaacgtcgtgactgggaaaaccctggcgttacccaacttaatcgccttgcag
cacatccccctttcgccagctggcgtaatagcgaagaggcccgcaccgatcgcccttcccaaca
gttgcgcagcctgaatggcgaatggcagcttggctgttttggcggatgagataagattttcagc
ctgatacagattaaatcagaacgcagaagcggtctgataaaacagaatttgcctggcggcagta
gcgcggtggtcccacctgaccccatgccgaactcagaagtgaaacgccgtagcgccgatggtag
tgtggggtctccccatgcgagagtagggaactgccaggcatcaaataaaacgaaaggctcagtc
gaaagactgggcctttcgttttatctgttgtttgtcggtgaacgctctcctgagtaggacaaat
ccgccgggagcggatttgaacgttgcgaagcaacggcccggagggtggcgggcaggacgcccgc
cataaactgccaggcatcaaattaagcagaaggccatcctgacggatggcctttttgcgtttct
acaaactctttttgtttatttttctaaatacattcaaatatgtatccgctcatgagacaataac
cctgataaatgcttcaataatattgaaaaaggaagagtatgagtattcaacatttccgtgtcgc

ccttattcccttttttgcggcattttgccttcctgtttttgctcacccagaaacgctggtgaaa
gtaaaagatgctgaagatcagttgggtgcacgagtgggttacatcgaactggatctcaacagcg
gtaagatccttgagagttttcgccccgaagaacgttctccaatgatgagcacttttaaagttct
gctatgtggcgcggtattatcccgtgttgacgccgggcaagagcaactcggtcgccgcatacac
tattctcagaatgacttggttgagtactcaccagtcacagaaaagcatcttacggatggcatga
cagtaagagaattatgcagtgctgccataaccatgagtgataacactgcggccaacttacttct
gacaacgatcggaggaccgaaggagctaaccgcttttttgcacaacatgggggatcatgtaact
cgccttgatcgttgggaaccggagctgaatgaagccataccaaacgacgagcgtgacaccacga
tgcctgtagcaatggcaacaacgttgcgcaaactattaactggcgaactacttactctagcttc
ccggcaacaattaatagactggatggaggcggataaagttgcaggaccacttctgcgctcggcc
cttccggctggctggtttattgctgataaatctggagccggtgagcgtgggtctcgcggtatca
ttgcagcactggggccagatggtaagccctcccgtatcgtagttatctacacgacggggagtca
ggcaactatggatgaacgaaatagacagatcgctgagataggtgcctcactgattaagcattgg
taactgtcagaccaagtttactcatatatactttagattgatttaccccggttgataatcagaa
aagccccaaaaacaggaagattgtataagcaaatatttaaattgtaaacgttaatattttgtta
aaattcgcgttaaatttttgttaaatcagctcattttttaaccaataggccgaaatcggcaaaa
tcccttataaatcaaaagaatagaccgagatagggttgagtgttgttccagtttggaacaagag
tccactattaaagaacgtggactccaacgtcaaagggcgaaaaaccgtctatcagggcgatggc
ccactacgtgaaccatcacccaaatcaagttttttggggtcgaggtgccgtaaagcactaaatc
ggaaccctaaagggagcccccgatttagagcttgacggggaaagccggcgaacgtggcgagaaa
ggaagggaagaaagcgaaaggagcgggcgctagggcgctggcaagtgtagcggtcacgctgcgc
gtaaccaccacacccgccgcgcttaatgcgccgctacagggcgcgtaaaaggatctaggtgaag
atcctttttgataatctcatgaccaaaatcccttaacgtgagttttcgttccactgagcgtcag
accccgtagaaaagatcaaaggatcttcttgagatcctttttttctgcgcgtaatctgctgctt
gcaaacaaaaaaaccaccgctaccagcggtggtttgtttgccggatcaagagctaccaactctt
tttccgaaggtaactggcttcagcagagcgcagataccaaatactgtccttctagtgtagccgt
agttaggccaccacttcaagaactctgtagcaccgcctacatacctcgctctgctaatcctgtt
accagtggctgctgccagtggcgataagtcgtgtcttaccgggttggactcaagacgatagtta
ccggataaggcgcagcggtcgggctgaacggggggttcgtgcacacagcccagcttggagcgaa
cgacctacaccgaactgagatacctacagcgtgagctatgagaaagcgccacgcttcccgaagg
gagaaaggcggacaggtatccggtaagcggcagggtcggaacaggagagcgcacgagggagctt
ccagggggaaacgcctggtatctttatagtcctgtcgggtttcgccacctctgacttgagcgtc
gatttttgtgatgctcgtcaggggggcggagcctatggaaaaacgccagcaacgcggcctttttt
acggttcctggccttttgctggccttttgctcacatgttctttcctgcgttatcccctgattct
gtggataaccgtattaccgcctttgagtgagctgataccgctcgccgcagccgaacgaccgagc
gcagcgagtcagtgagcgaggaagcggaagagcgcctgatgcggtattttctccttacgcatct
gtgcggtatttcacaccgcatatatggtgcactctcagtacaatctgctctgatgccgcatagt
taagccagtatacactccgctatcgctacgtgactgggtcatggctgcgccccgacacccgcca
acacccgctgacgcgccctgacgggcttgtctgctcccggcatccgcttacagacaagctgtga
ccgtctccgggagctgcatgtgtcagaggttttcaccgtcatcaccgaaacgcgcgaggcagct
gcggtaaagctcatcagcgtggtcgtgcagcgattcacagatgtctgcctgttcatccgcgtcc
agctcgttgagtttctccagaagcgttaatgtctggcttctgataaagcgggccatgttaaggg
cggttttttcctgtttggtcactgatgcctccgtgtaagggggatttctgttcatgggggtaat
gataccgatgaaacgagagaggatgctcacgatacgggttactgatgatgaacatgcccggtta
ctggaacgttgtgagggtaaacaactggcggtatggatgcggcgggaccagagaaaaatcactc
agggtcaatgccagcgcttcgttaatacagatgtaggtgttccacagggtagccagcagcatcc
tgcgatgcagatccggaacataatggtgcagggcgctgacttccgcgtttccagactttacgaa

acacggaaaccgaagaccattcatgttgttgctcaggtcgcagacgttttgcagcagcagtcgc
ttcacgttcgctcgcgtatcggtgattcattctgctaaccagtaaggcaaccccgccagcctag
ccgggtcctcaacgacaggagcacgatcatgcgcacccgtggccaggacccaacgctgcccgaa
att-3´

pMAL-TEV-Gal3(111)-E3-His

5´-ccgacaccatcgaatggtgcaaaacctttcgcggtatggcatgatagcgcccggaagagag
tcaattcagggtggtgaatgtgaaaccagtaacgttatacgatgtcgcagagtatgccggtgtc
tcttatcagaccgtttcccgcgtggtgaaccaggccagccacgtttctgcgaaaacgcgggaaa
aagtggaagcggcgatggcggagctgaattacattcccaaccgcgtggcacaacaactggcggg
caaacagtcgttgctgattggcgttgccacctccagtctggccctgcacgcgccgtcgcaaatt
gtcgcggcgattaaatctcgcgccgatcaactgggtgccagcgtggtggtgtcgatggtagaac
gaagcggcgtcgaagcctgtaaagcggcggtgcacaatcttctcgcgcaacgcgtcagtgggct
gatcattaactatccgctggatgaccaggatgccattgctgtggaagctgcctgcactaatgtt
ccggcgttatttcttgatgtctctgaccagacacccatcaacagtattattttctcccatgaag
acggtacgcgactgggcgtggagcatctggtcgcattgggtcaccagcaaatcgcgctgttagc
gggcccattaagttctgtctcggcgcgtctgcgtctggctggctggcataaatatctcactcgc
aatcaaattcagccgatagcggaacgggaaggcgactggagtgccatgtccggttttcaacaaa
ccatgcaaatgctgaatgagggcatcgttcccactgcgatgctggttgccaacgatcagatggc
gctgggcgcaatgcgcgccattaccgagtccgggctgcgcgttggtgcggatatctcggtagtg
ggatacgacgataccgaagacagctcatgttatatcccgccgttaaccaccatcaaacaggatt
ttcgcctgctggggcaaaccagcgtggaccgcttgctgcaactctctcagggccaggcggtgaa
gggcaatcagctgttgcccgtctcactggtgaaaagaaaaaccaccctggcgcccaatacgcaa
accgcctctccccgcgcgttggccgattcattaatgcagctggcacgacaggtttcccgactgg
aaagcgggcagtgagcgcaacgcaattaatgtaagttagctcactcattaggcacaattctcat
gtttgacagcttatcatcgactgcacggtgcaccaatgcttctggcgtcaggcagccatcggaa
gctgtggtatggctgtgcaggtcgtaaatcactgcataattcgtgtcgctcaaggcgcactccc
gttctggataatgttttttgcgccgacatcataacggttctggcaaatattctgaaatgagctg
ttgacaattaatcatcggctcgtataatgtgtggaattgtgagcggataacaatttcacacagg
aaacagccagtccgtttaggtgttttcacgagcacttcaccaacaaggaccatagcatatgaaa
atcgaagaaggtaaactggtaatctggattaacggcgataaaggctataacggtctcgctgaag
tcggtaagaaattcgagaaagataccggaattaaagtcaccgttgagcatccggataaactgga
agagaaattcccacaggttgcggcaactggcgatggccctgacattatcttctggcacacgac
cgctttggtggctacgctcaatctggcctgttggctgaaatcaccccggacaaagcgttccagg
acaagctgtatccgtttacctgggatgccgtacgttacaacggcaagctgattgcttacccgat
cgctgttgaagcgttatcgctgatttataacaaagatctgctgccgaacccgccaaaaacctgg
gaagagatcccggcgctggataaagaactgaaagcgaaaggtaagagcgcgctgatgttcaacc
tgcaagaaccgtacttcacctggccgctgattgctgctgacggggttatgcgttcaagtatga
aaacggcaagtacgacattaaagacgtgggcgtggataacgctggcgcgaaagcgggtctgacc
ttcctggttgacctgattaaaaacaaacacatgaatgcagacaccgattactccatcgcagaag
ctgcctttaataaaggcgaaacagcgatgaccatcaacggcccgtgggcatggtccaacatcga
caccagcaaagtgaattatggtgtaacggtactgccgaccttcaagggtcaaccatccaaaccg
ttcgttggcgtgctgagcgcaggtattaacgccgccagtccgaacaaagagctggcaaaagagt

tcctcgaaaactatctgctgactgatgaaggtctggaagcggttaataaagacaaaccgctggg
tgccgtagcgctgaagtcttacgaggaagagttggcgaaagatccacgtattgccgccactatg
gaaaacgcccagaaaggtgaaatcatgccgaacatcccgcagatgtccgctttctggtatgccg
tgcgtactgcggtgatcaacgccgccagcggtcgtcagactgtcgatgaagccctgaaagacgc
gcagactaattcgagctcgaacaacaacaacaataacaataacaacaacctcgggagctcggag
aatctttattttcagggcggatccgctgggccactgattgtgccttataacctgcctttgcctg
gggagtggtgcctcgcatgctgataacaattctgggcacggtgaagcccaatgcaaacagaat
tgctttagatttccaaagagggaatgatgttgccttccactttaacccacgcttcaatgagaac
aacaggagagtcattgtttgcaatacaaagctggataataactggggaagggaagaaagacagt
cggttttcccatttgaaagtgggaaaccattcaaaatacaagtactggttgaacctgaccactt
caaggttgcagtgaatgatgctcacttgttgcagtacaatcatcgggttaaaaaactcaatgaa
atcagcaaactgggaatttctggtgacatagacctcaccagtgcttcatataccatgatagtcg
acggcggaggaggatctggcggaggaggatctctgcaggccatggaaatcgccgctctagaaaa
agagatcgctgctctggagaaggagattgccgcccttgagaagggcggcgcggccgccggcgga
ggaggatctcatcatcaccaccaccattaagcttggcactggccgtcgttttacaacgtcgtga
ctgggaaaaccctggcgttacccaacttaatcgccttgcagcacatcccccttcgccagctgg
cgtaatagcgaagaggcccgcaccgatcgcccttcccaacagttgcgcagcctgaatggcgaat
ggcagcttggctgttttggcggatgagataagattttcagcctgatacagattaaatcagaacg
cagaagcggtctgataaaacagaatttgcctggcggcagtagcgcggtggtcccacctgacccc
atgccgaactcagaagtgaaacgccgtagcgccgatggtagtgtggggtctccccatgcgagag
tagggaactgccaggcatcaaataaaacgaaaggctcagtcgaaagactgggcctttcgtttta
tctgttgtttgtcggtgaacgctctcctgagtaggacaaatccgccgggagcggatttgaacgt
tgcgaagcaacggcccggagggtggcgggcaggacgcccgccataaactgccaggcatcaaatt
aagcagaaggccatcctgacggatggcctttttgcgtttctacaaactctttttgtttattttt
ctaaatacattcaaatatgtatccgctcatgagacaataaccctgataaatgcttcaataatat
tgaaaaaggaagagtatgagtattcaacatttccgtgtcgcccttattcccttttttgcggcat
tttgccttcctgtttttgctcacccagaaacgctggtgaaagtaaaagatgctgaagatcagtt
gggtgcacgagtgggttacatcgaactggatctcaacagcggtaagatccttgagagttttcgc
cccgaagaacgttctccaatgatgagcacttttaaagttctgctatgtggcgcggtattatccc
gtgttgacgccgggcaagagcaactcggtcgccgcatacactattctcagaatgacttggttga
gtactcaccagtcacagaaaagcatcttacggatggcatgacagtaagagaattatgcagtgct
gccataaccatgagtgataacactgcggccaacttacttctgacaacgatcggaggaccgaagg
agctaaccgcttttttgcacaacatgggggatcatgtaactcgccttgatcgttgggaaccgga
gctgaatgaagccataccaaacgacgagcgtgacaccacgatgcctgtagcaatggcaacaacg
ttgcgcaaactattaactggcgaactacttactctagcttcccggcaacaattaatagactgga
tggaggcggataaagttgcaggaccacttctgcgctcggcccttccggctggctggtttattgc
tgataaatctggagccggtgagcgtgggtctcgcggtatcattgcagcactggggccagatggt
aagccctcccgtatcgtagttatctacacgacggggagtcaggcaactatggatgaacgaaata
gacagatcgctgagataggtgcctcactgattaagcattggtaactgtcagaccaagtttactc
atatatactttagattgatttaccccggttgataatcagaaaagccccaaaaacaggaagattg
tataagcaaatatttaaattgtaaacgttaatattttgttaaaattcgcgttaaatttttgtta
aatcagctcattttttaaccaataggccgaaatcggcaaaatcccttataaatcaaaagaatag
accgagatagggttgagtgttgttccagtttggaacaagagtccactattaaagaacgtggact
ccaacgtcaaagggcgaaaaaccgtctatcagggcgatggcccactacgtgaaccatcacccaa
atcaagttttttggggtcgaggtgccgtaaagcactaaatcggaaccctaaagggagcccccga
tttagagcttgacggggaaagccggcgaacgtggcgagaaaggaagggaagaaagcgaaaggag
cgggcgctagggcgctggcaagtgtagcggtcacgctgcgcgtaaccaccacacccgccgcgct

taatgcgccgctacagggcgcgtaaaaggatctaggtgaagatcctttttgataatctcatgac
caaaatcccttaacgtgagttttcgttccactgagcgtcagaccccgtagaaaagatcaaagga
tcttcttgagatcctttttttctgcgcgtaatctgctgcttgcaaacaaaaaaaccaccgctac
cagcggtggtttgtttgccggatcaagagctaccaactctttttccgaaggtaactggcttcag
cagagcgcagataccaaatactgtccttctagtgtagccgtagttaggccaccacttcaagaac
tctgtagcaccgcctacatacctcgctctgctaatcctgttaccagtggctgctgccagtggcg
ataagtcgtgtcttaccgggttggactcaagacgatagttaccggataaggcgcagcggtcggg
ctgaacggggggttcgtgcacacagcccagcttggagcgaacgacctacaccgaactgagatac
ctacagcgtgagctatgagaaagcgccacgcttcccgaagggagaaaggcggacaggtatccgg
taagcggcagggtcggaacaggagagcgcacgagggagcttccagggggaaacgcctggtatct
ttatagtcctgtcgggtttcgccacctctgacttgagcgtcgatttttgtgatgctcgtcaggg
gggcggagcctatggaaaaacgccagcaacgcggcctttttacggttcctggccttttgctggc
cttttgctcacatgttctttcctgcgttatcccctgattctgtggataaccgtattaccgcctt
tgagtgagctgataccgctcgccgcagccgaacgaccgagcgcagcgagtcagtgagcgaggaa
gcggaagagcgcctgatgcggtattttctccttacgcatctgtgcggtatttcacaccgcatat
atggtgcactctcagtacaatctgctctgatgccgcatagttaagccagtatacactccgctat
cgctacgtgactgggtcatggctgcgccccgacacccgccaacacccgctgacgcgccctgacg
ggcttgtctgctcccggcatccgcttacagacaagctgtgaccgtctccgggagctgcatgtgt
cagaggttttcaccgtcatcaccgaaacgcgcgaggcagctgcggtaaagctcatcagcgtggt
cgtgcagcgattcacagatgtctgcctgttcatccgcgtccagctcgttgagtttctccagaag
cgttaatgtctggcttctgataaagcgggccatgttaagggcggttttttcctgtttggtcact
gatgcctccgtgtaagggggatttctgttcatgggggtaatgataccgatgaaacgagagagga
tgctcacgatacgggttactgatgatgaacatgcccggttactggaacgttgtgagggtaaaca
actggcggtatggatgcggcgggaccagagaaaaatcactcagggtcaatgccagcgcttcgtt
aatacagatgtaggtgttccacagggtagccagcagcatcctgcgatgcagatccggaacataa
tggtgcagggcgctgacttccgcgtttccagactttacgaaacacggaaaccgaagaccattca
tgttgttgctcaggtcgcagacgttttgcagcagcagtcgcttcacgttcgctcgcgtatcggt
gattcattctgctaaccagtaaggcaaccccgccagcctagccgggtcctcaacgacaggagca
cgatcatgcgcacccgtggccaggacccaacgctgcccgaaatt-3´

pMAL-TEV-mSA2-K3-His

5´-ccgacaccatcgaatggtgcaaaacctttcgcggtatggcatgatagcgcccggaagagag
tcaattcagggtggtgaatgtgaaaccagtaacgttatacgatgtcgcagagtatgccggtgtc
tcttatcagaccgtttcccgcgtggtgaaccaggccagccacgtttctgcgaaaacgcgggaaa
aagtggaagcggcgatggcggagctgaattacattcccaaccgcgtggcacaacaactggcggg
caaacagtcgttgctgattggcgttgccacctccagtctggccctgcacgcgccgtcgcaaatt
gtcgcggcgattaaatctcgcgccgatcaactgggtgccagcgtggtggtgtcgatggtagaac
gaagcggcgtcgaagcctgtaaagcggcggtgcacaatcttctcgcgcaacgcgtcagtgggct
gatcattaactatccgctggatgaccaggatgccattgctgtggaagctgcctgcactaatgtt
ccggcgttatttcttgatgtctctgaccagacacccatcaacagtattattttctcccatgaag
acggtacgcgactgggcgtggagcatctggtcgcattgggtcaccagcaaatcgcgctgttagc
gggcccattaagttctgtctcggcgcgtctgcgtctggctggctggcataaatatctcactcgc
aatcaaattcagccgatagcggaacgggaaggcgactggagtgccatgtccggttttcaacaaa

ccatgcaaatgctgaatgagggcatcgttcccactgcgatgctggttgccaacgatcagatggc
gctgggcgcaatgcgcgccattaccgagtccgggctgcgcgttggtgcggatatctcggtagtg
ggatacgacgataccgaagacagctcatgttatatcccgccgttaaccaccatcaaacaggatt
ttcgcctgctggggcaaaccagcgtggaccgcttgctgcaactctctcagggccaggcggtgaa
gggcaatcagctgttgcccgtctcactggtgaaaagaaaaaccaccctggcgcccaatacgcaa
accgcctctccccgcgcgttggccgattcattaatgcagctggcacgacaggtttcccgactgg
aaagcgggcagtgagcgcaacgcaattaatgtaagttagctcactcattaggcacaattctcat
gtttgacagcttatcatcgactgcacggtgcaccaatgcttctggcgtcaggcagccatcggaa
gctgtggtatggctgtgcaggtcgtaaatcactgcataattcgtgtcgctcaaggcgcactccc
gttctggataatgttttttgcgccgacatcataacggttctggcaaatattctgaaatgagctg
ttgacaattaatcatcggctcgtataatgtgtggaattgtgagcggataacaatttcacacagg
aaacagccagtccgtttaggtgttttcacgagcacttcaccaacaaggaccatagcatatgaaa
atcgaagaaggtaaactggtaatctggattaacggcgataaaggctataacggtctcgctgaag
tcggtaagaaattcgagaaagataccggaattaaagtcaccgttgagcatccggataaactgga
agagaaattcccacaggttgcggcaactggcgatggccctgacattatcttctggcacacgac
cgctttggtggctacgctcaatctggcctgttggctgaaatcaccccggacaaagcgttccagg
acaagctgtatccgtttacctgggatgccgtacgttacaacggcaagctgattgcttacccgat
cgctgttgaagcgttatcgctgatttataacaaagatctgctgccgaacccgccaaaaacctgg
gaagagatcccggcgctggataaagaactgaaagcgaaaggtaagagcgcgctgatgttcaacc
tgcaagaaccgtacttcacctggccgctgattgctgctgacggggttatgcgttcaagtatga
aaacggcaagtacgacattaaagacgtgggcgtggataacgctggcgcgaaagcgggtctgacc
ttcctggttgacctgattaaaaacaaacacatgaatgcagacaccgattactccatcgcagaag
ctgcctttaataaaggcgaaacagcgatgaccatcaacggcccgtgggcatggtccaacatcga
caccagcaaagtgaattatggtgtaacggtactgccgaccttcaagggtcaaccatccaaaccg
ttcgttggcgtgctgagcgcaggtattaacgccgccagtccgaacaaagagctggcaaaagagt
tcctcgaaaactatctgctgactgatgaaggtctggaagcggttaataaagacaaaccgctggg
tgccgtagcgctgaagtcttacgaggaagagttggcgaaagatccacgtattgccgccactatg
gaaaacgcccagaaaggtgaaatcatgccgaacatcccgcagatgtccgctttctggtatgccg
tgcgtactgcggtgatcaacgccgccagcggtcgtcagactgtcgatgaagccctgaaagacgc
gcagactaattcgagctcgaacaacaacaacaataacaataacaacaacctcgggagctcggag
aatctttattttcagggcggatccgcggaagcgggtatcaccggcacgtggtacaaccagcatg
gttctaccttcaccgttaccgcgggtgcggacggtaacctgaccggtcagtacgaaaaccgtgc
gcagggcactggttgccagaactctccgtacaccctgaccggtcgttacaacggtaccaaactg
gaatggcgtgttgaatggaacaactctaccgaaaactgccactctcgtaccgaatggcgtggtc
agtaccagggtggtgcggaagcgcgtatcaacacccagtggaacctgacctacgaaggtggttc
tggtccggcgaccgaacagggtcaggacaccttcaccaaagttaaaccgtctgcggcgtctgga
aacagcagttccatggccatgaagatcgccgccctgaaggagaagatcgccgccctgaaggaga
agatcgccgcccttaaggagggcggcgcggccgccggcggaggaggatctcatcatcaccacca
ccattaagcttggcactggccgtcgttttacaacgtcgtgactgggaaaaccctggcgttaccc
aacttaatcgccttgcagcacatccccctttcgccagctggcgtaatagcgaagaggcccgcac
cgatcgcccttcccaacagttgcgcagcctgaatggcgaatggcagcttggctgttttggcgga
tgagataagattttcagcctgatacagattaaatcagaacgcagaagcggtctgataaaacaga
atttgcctggcggcagtagcgcggtggtcccacctgaccccatgccgaactcagaagtgaaacg
ccgtagcgccgatggtagtgtggggtctccccatgcgagagtagggaactgccaggcatcaaat
aaaacgaaaggctcagtcgaaagactgggcctttcgttttatctgttgtttgtcggtgaacgct
ctcctgagtaggacaaatccgccgggagcggatttgaacgttgcgaagcaacggcccggagggt
ggcgggcaggacgcccgccataaactgccaggcatcaaattaagcagaaggccatcctgacgga

tggccttttgcgtttctacaaactcttttgtttatttttctaaatacattcaaatatgtatc
cgctcatgagacaataaccctgataaatgcttcaataatattgaaaaaggaagagtatgagtat
tcaacatttccgtgtcgcccttattcccttttttgcggcattttgccttcctgtttttgctcac
ccagaaacgctggtgaaagtaaaagatgctgaagatcagttgggtgcacgagtgggttacatcg
aactggatctcaacagcggtaagatccttgagagttttcgccccgaagaacgttctccaatgat
gagcacttttaaagttctgctatgtggcgcggtattatcccgtgttgacgccgggcaagagcaa
ctcggtcgccgcatacactattctcagaatgacttggttgagtactcaccagtcacagaaaagc
atcttacggatggcatgacagtaagagaattatgcagtgctgccataaccatgagtgataacac
tgcggccaacttacttctgacaacgatcggaggaccgaaggagctaaccgcttttttgcacaac
atgggggatcatgtaactcgccttgatcgttgggaaccggagctgaatgaagccataccaaacg
acgagcgtgacaccacgatgcctgtagcaatggcaacaacgttgcgcaaactattaactggcga
actacttactctagcttcccggcaacaattaatagactggatggaggcggataaagttgcagga
ccacttctgcgctcggcccttccggctggctggtttattgctgataaatctggagccggtgagc
gtgggtctcgcggtatcattgcagcactggggccagatggtaagccctcccgtatcgtagttat
ctacacgacggggagtcaggcaactatggatgaacgaaatagacagatcgctgagataggtgcc
tcactgattaagcattggtaactgtcagaccaagtttactcatatatactttagattgatttac
cccggttgataatcagaaaagccccaaaaacaggaagattgtataagcaaatatttaaattgta
aacgttaatattttgttaaaattcgcgttaaatttttgttaaatcagctcattttttaaccaat
aggccgaaatcggcaaaatcccttataaatcaaaagaatagaccgagatagggttgagtgttgt
tccagtttggaacaagagtccactattaaagaacgtggactccaacgtcaaagggcgaaaaacc
gtctatcagggcgatggcccactacgtgaaccatcacccaaatcaagttttttggggtcgaggt
gccgtaaagcactaaatcggaaccctaaagggagcccccgatttagagcttgacggggaaagcc
ggcgaacgtggcgagaaaggaagggaagaaagcgaaaggagcgggcgctagggcgctggcaagt
gtagcggtcacgctgcgcgtaaccaccacacccgccgcgcttaatgcgccgctacagggcgcgt
aaaaggatctaggtgaagatcctttttgataatctcatgaccaaaatcccttaacgtgagtttt
cgttccactgagcgtcagaccccgtagaaaagatcaaaggatcttcttgagatcctttttttct
gcgcgtaatctgctgcttgcaaacaaaaaaaccaccgctaccagcggtggtttgtttgccggat
caagagctaccaactctttttccgaaggtaactggcttcagcagagcgcagataccaaatactg
tccttctagtgtagccgtagttaggccaccacttcaagaactctgtagcaccgcctacatacct
cgctctgctaatcctgttaccagtggctgctgccagtggcgataagtcgtgtcttaccgggttg
gactcaagacgatagttaccggataaggcgcagcggtcgggctgaacggggggttcgtgcacac
agcccagcttggagcgaacgacctacaccgaactgagatacctacagcgtgagctatgagaaag
cgccacgcttcccgaagggagaaaggcggacaggtatccggtaagcggcagggtcggaacagga
gagcgcacgagggagcttccagggggaaacgcctggtatctttatagtcctgtcgggtttcgcc
acctctgacttgagcgtcgatttttgtgatgctcgtcaggggggcggagcctatggaaaaacgc
cagcaacgcggcctttttacggttcctggccttttgctggccttttgctcacatgttctttcct
gcgttatcccctgattctgtggataaccgtattaccgcctttgagtgagctgataccgctcgcc
gcagccgaacgaccgagcgcagcgagtcagtgagcgaggaagcggaagagcgcctgatgcggta
ttttctccttacgcatctgtgcggtatttcacaccgcatatatggtgcactctcagtacaatct
gctctgatgccgcatagttaagccagtatacactccgctatcgctacgtgactgggtcatggct
gcgccccgacacccgccaacacccgctgacgcgccctgacgggcttgtctgctcccggcatccg
cttacagacaagctgtgaccgtctccgggagctgcatgtgtcagaggttttcaccgtcatcacc
gaaacgcgcgaggcagctgcggtaaagctcatcagcgtggtcgtgcagcgattcacagatgtct
gcctgttcatccgcgtccagctcgttgagtttctccagaagcgttaatgtctggcttctgataa
agcgggccatgttaagggcggttttttcctgtttggtcactgatgcctccgtgtaagggggatt
tctgttcatgggggtaatgataccgatgaaacgagagaggatgctcacgatacgggttactgat
gatgaacatgcccggttactggaacgttgtgagggtaaacaactggcggtatggatgcggcggg

accagagaaaaatcactcagggtcaatgccagcgcttcgttaatacagatgtaggtgttccaca
gggtagccagcagcatcctgcgatgcagatccggaacataatggtgcagggcgctgacttccgc
gtttccagactttacgaaacacggaaaccgaagaccattcatgttgttgctcaggtcgcagacg
ttttgcagcagcagtcgcttcacgttcgctcgcgtatcggtgattcattctgctaaccagtaag
gcaaccccgccagcctagccgggtcctcaacgacaggagcacgatcatgcgcacccgtggccag
gacccaacgctgcccgaaatt-3´

3. Figure S1. *Expression and purification of the recombinant protein used in this article.* Novagen's Rosetta Blue™ cells bearing plasmids pMAL-TEV-GAL3-His (A), pMAL-TEV-GAL3(111)-His (B), pMBP-mSA2-K_3-His (C) or pMAL-TEV-GAL3(111)-E_3-His (D) were grown in LB Broth medium until OD600 = 0.5 and then, cells were incubated with 0.5mM IPTG to induce the protein expression at 30°C for an additional period of 8 h and then digested or not with TEV protease as described in the Material and Methods section. Recombinant proteins were purified by affinity chromatography using an IMAC column. MWM, protein molecular weight marker, EC, crude extract, SN, supernatant; TEV; fraction after digestion with TEV protease; FT, Flow-through the affinity column; AF, affinity chromatography eluted fraction. Asterisks mark the expected molecular weight of the recombinant proteins.

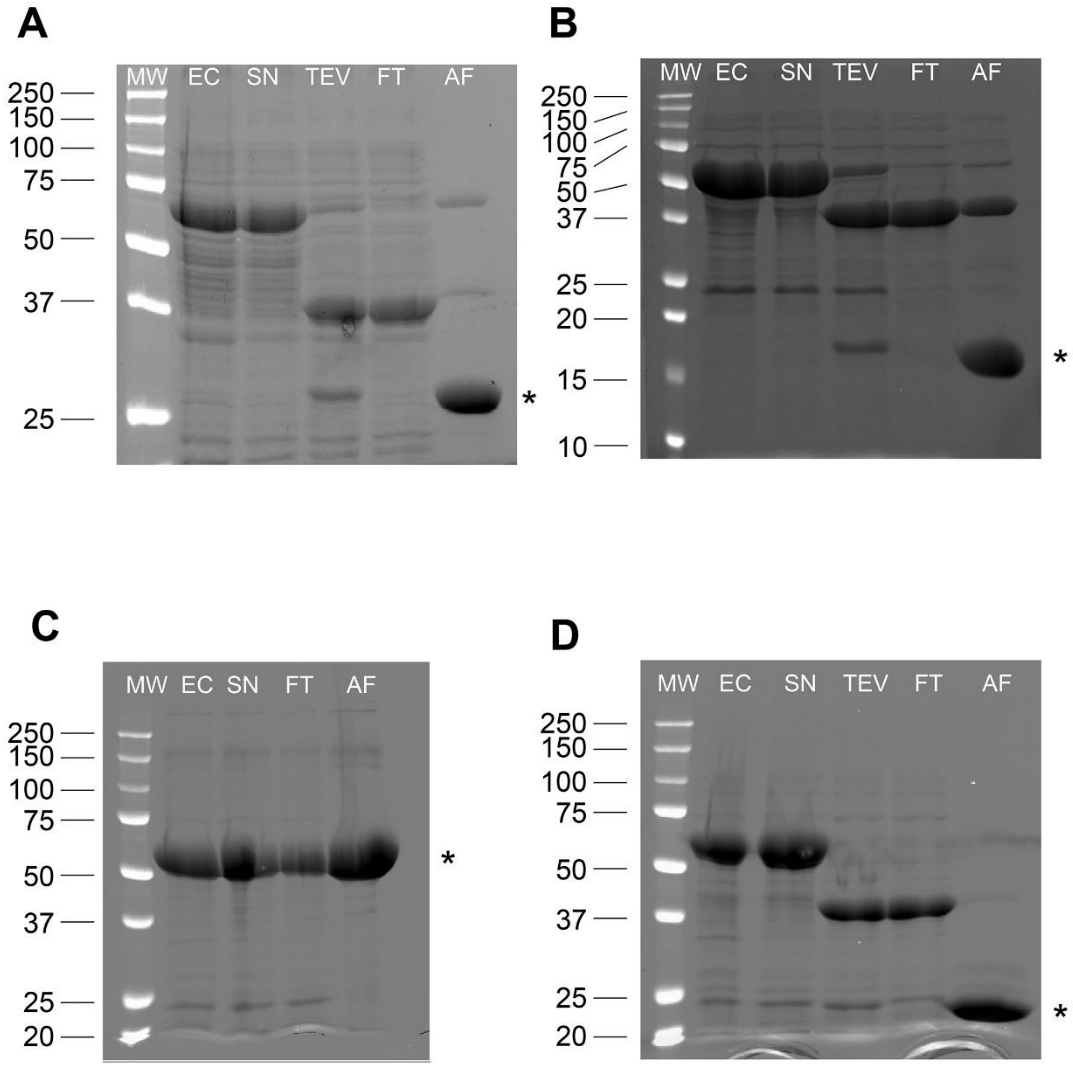

4. **Figure S2.** *Synthesis of biotin-doxorubicin.*

A D-Biotinol was obtained by reduction of D-Biotin with $LiAlH_4$ (A) according to the methodology previously reported.[1]

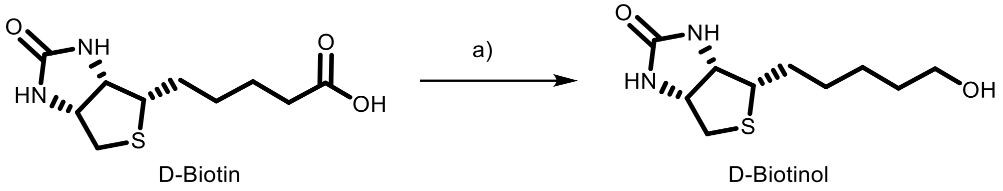

(a)(1) MeOH, H_2SO_4, RT, overnight (2) $LiAlH_4$, THF, 3 h, RT.

D-biotinyl vinylsulfonate (B) was prepared by adding a solution of D-Biotinol (55 mg) and Et_3N (100mL) in anhydrous CH_2Cl_2 (2mL) to a solution of 2-chloroethanesulfonyl chloride (40mL) in anhydrous CH_2Cl_2 (2mL), cooled in a water-ice bath. The mixture was stirred for 1 h at 0–4 °C under an inert atmosphere. The solvent was removed under reduced pressure. The crude residue was purified by column chromatography (Cl_2CH_2/MeOH, 20:1) to afford a white solid 26mg, yield 34%. ^{1}H NMR (400 MHz, CD_3CN, δ):6.69 (dd, *J* = 16.6, 10.1 Hz, 1H), 6.37 (d, *J* = 16.6 Hz, 1H), 6.23 (d, *J* = 10.1 Hz, 1H), 5.93 – 5.82 (m, 1H), 5.62 (s, 1H), 4.51 – 4.40 (m, 1H), 4.34 – 4.23 (m, 1H), 4.13 (t, *J* = 6.4 Hz, 2H), 3.61 (t, *J* = 6.7 Hz, 1H), 3.19 (ddd, *J* = 8.4, 6.4, 4.5 Hz, 1H), 2.91 (dd, *J* = 12.7, 5.0 Hz, 1H), 2.66 (d, *J* = 12.7 Hz, 1H), 1.87 – 1.34 (m, 8H).^{13}C NMR (101 MHz, CD_3CN, δ): 164.4, 133.2, 131.5, 72.2, 62.5, 60.7, 56.4, 41.1, 29.3, 29.2, 29.1, 26.0.

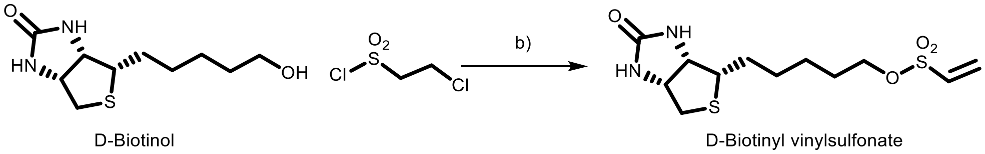

(b) Cl_2CH_2, Et_3N, 0ºC, 1hour.

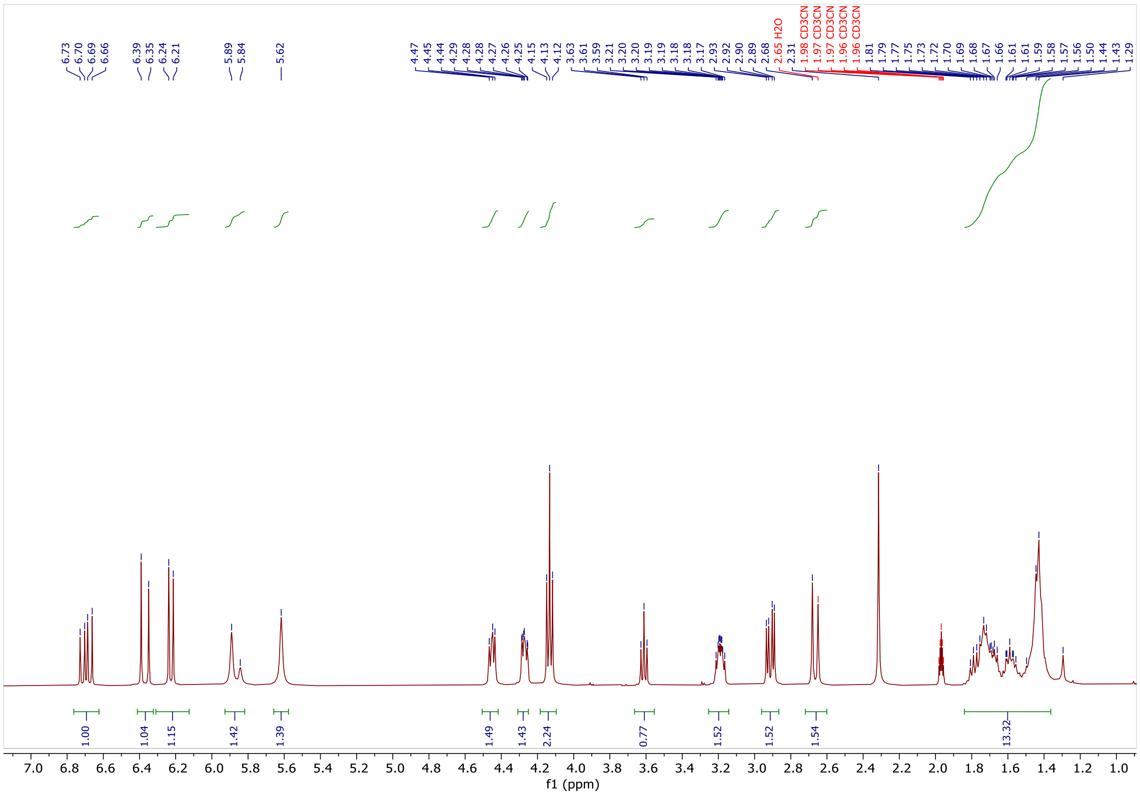

B ^{1}H NMR spectra of D-biotinyl vinylsulfonate

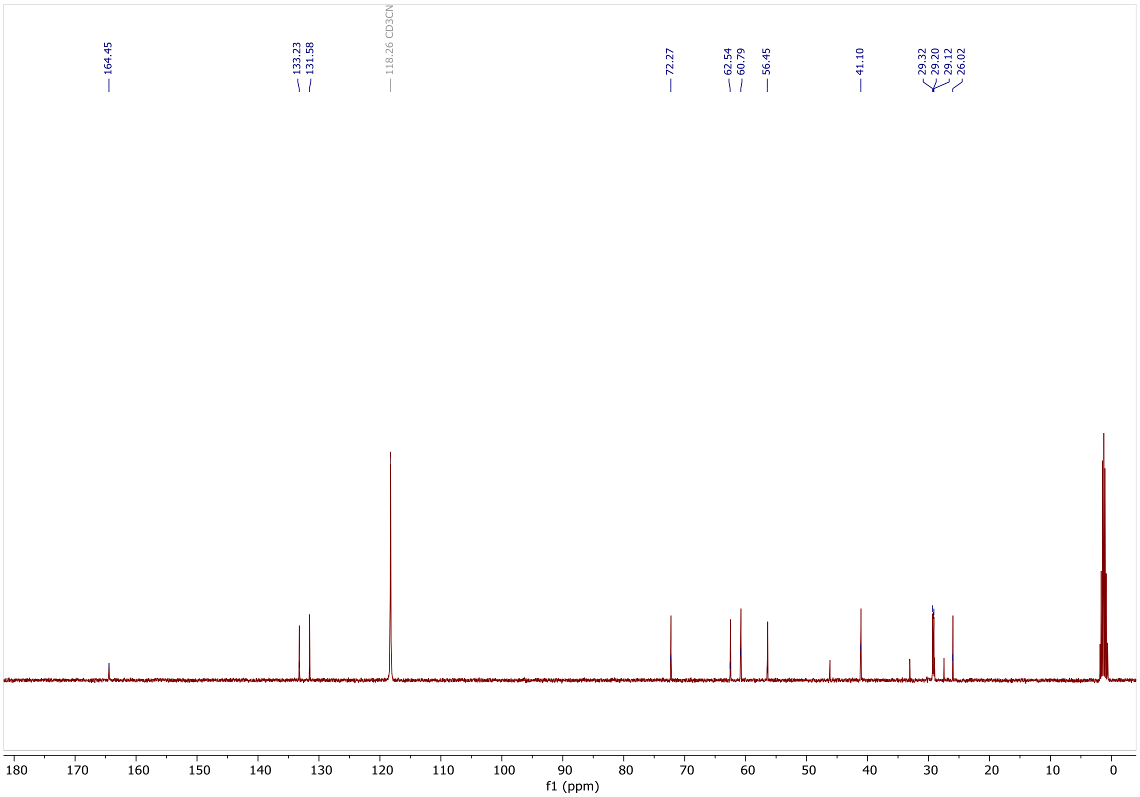

C ^{13}C NMR spectra of D-biotinyl vinylsulfonate

Biotin-Doxorubicin conjugate (D, E) was synthesized by dissolving 16.5 mg of biotinyl vinylsulfonate, 59.5 mg of doxorubicin, and 50 mL of Et3N in methanol (5 mL). The reaction was allowed to continue overnight, and after verifying the disappearance of biotinyl vinylsulfonate by TLC (ACN/H2O, 5:1), the solvent was evaporated, and the crude product was purified on a column using the same polarity. After lyophilization, 25 mg was obtained, with a yield of 54%.

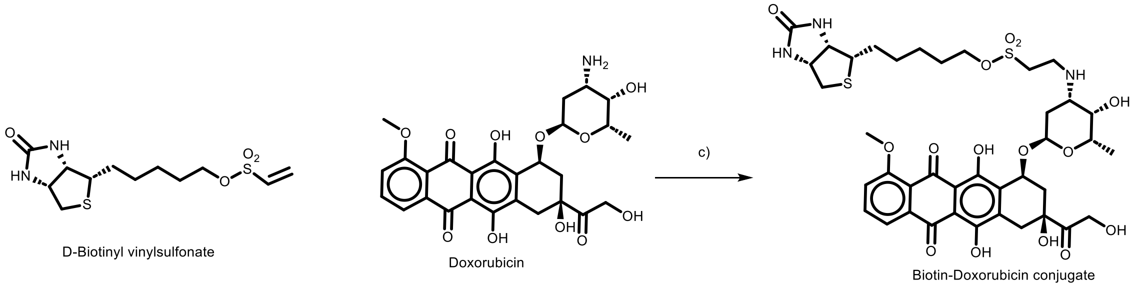

(c) Et3N, MeOH, rt.

D Synthetic strategy for the generation of biotin-doxorubicin conjugates

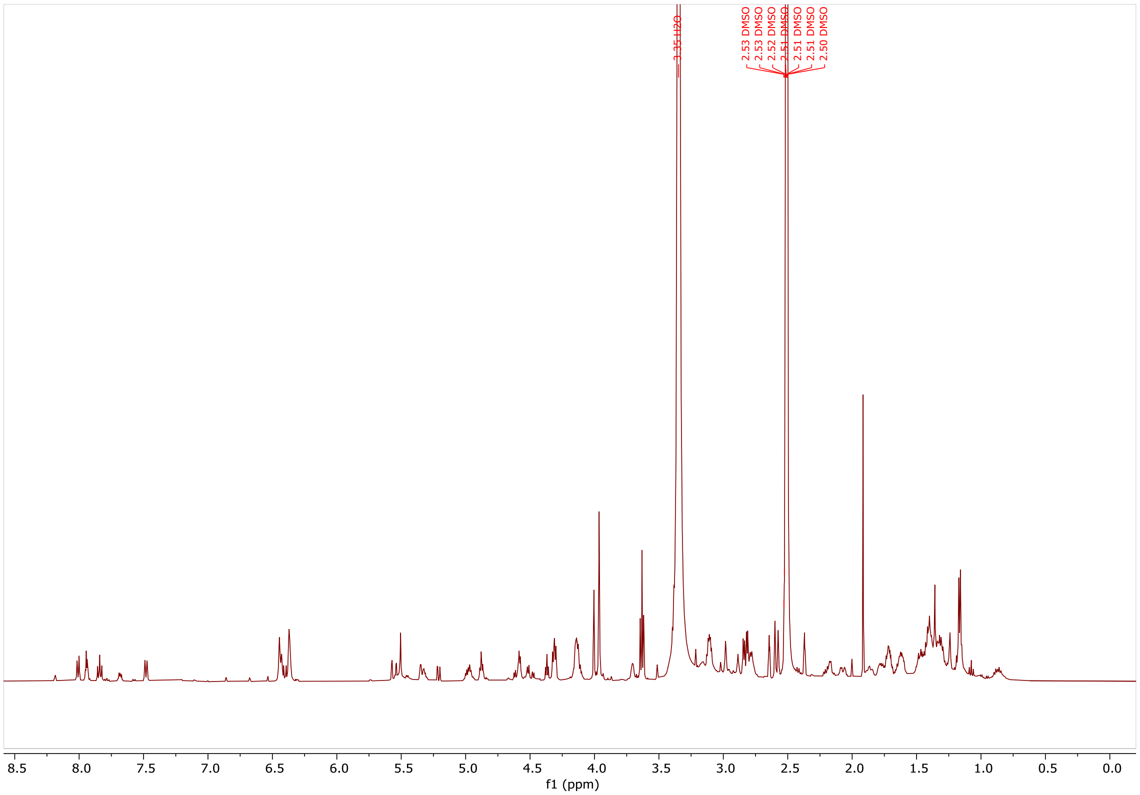

E ^{1}H NMR (DMSO-d6) spectra of Biotin-Doxorubicin conjugate

1. G. Giovannini, A. J. Hall and V. Gubala, *Talanta*, 2018, **188**, 448-453.

F *Release of DOX from b-DOX in the presence of reductors.* b-DOX was incubated in the presence of MBP-mSA2-K_3 protein (10 µg) for 10 min, and then nothing (1), β-mercaptoethanol (2), DTT (3), or reduced GSH (4) (10 µM final concentration) were added for an additional 30 min period. After incubation, samples were resolved by native electrophoresis in polyacrylamide gel containing TCE and the fluorescence associated to the DOX and protein content was detected.

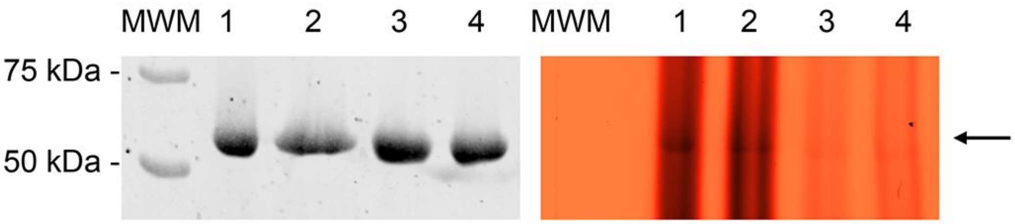

Figure S3. *Silencing of the expression of MUC1 in HeLa cells using CRISPR Cas9.* HeLa cells were transfected with the PX459-MUC1 (See Materials and Methods Section) using Lipofectamine 2000. The transfected cells were grown for 2 days and then selected with puromycin for an additional 2 days. The surviving cells were clonally diluted into 96-well plates to generate clonal populations from single cells. Western blotting was used to analyze isolated clones using an anti-MUC1 antibody. As a loading control, GAPDH expression was measured (antiGAPDH: hFAB™ Rhodamine Anti-GAPDH Primary Antibody, Cat. #12004167; Bio-Rad, Madrid, Spain). Clones #1 and #4 were pooled and used for the assays described in the article.

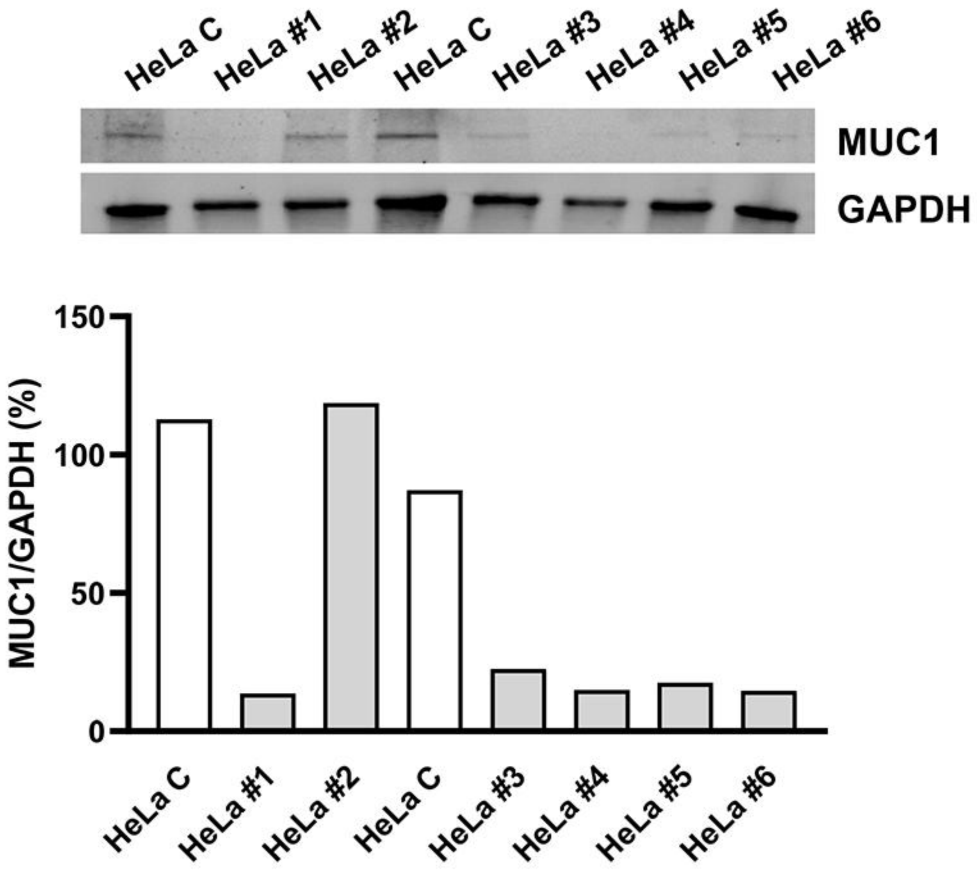

Figure S4. *Targeting the HER2 receptor in SKBR3 cells using a Coil-coiled system.* An expression vector coding for an anti HER2 ScFv antibody fused to an E3 motif has been generated by replacing the *Bam*HI/*Sal*I fragment coding for Gal3(111) for a BamHI/SalI fragment coding for the HER2 ScFv antibody described in. [2] The recombinant protein HER2 ScFv-E3 has been purified as described in the material and method section. It has been used to construct a coil-coiled delivery system termed [HER2-ScFv-E_3-MBP-mSA2-K_3] A.- Equimolecular amounts of HER2 ScFv-E_3 and MBP-mSA2-K_3 (700 pmol) were incubated at room temperature for 10 min. and then electrophoresed in TAE 0.8% agarose containing 5 µL/mL trichloroethanol. After electrophoresis, the gel was developed as described in the Materials and Methods section. B.- Targeting the capacity of biotin-ATTO647 bound to different ligands in cells expressing or not expressing the HER2 receptor. Uptake of biotin-ATTO647 bound to various proteins (10 µM) in HER2-positive cells (SKBR3) and negative cells (MDA-MB-231). The error bars represent S.E.M. of six replicates. *P<0.05 vs Biotin-ATTO647 treated cells. C.- Confocal images of SKBR3 and MDA-MB-231 cells incubated with Biotin-ATTO647~[HER2 ScFv-E3-MBP-mSA2-K3].

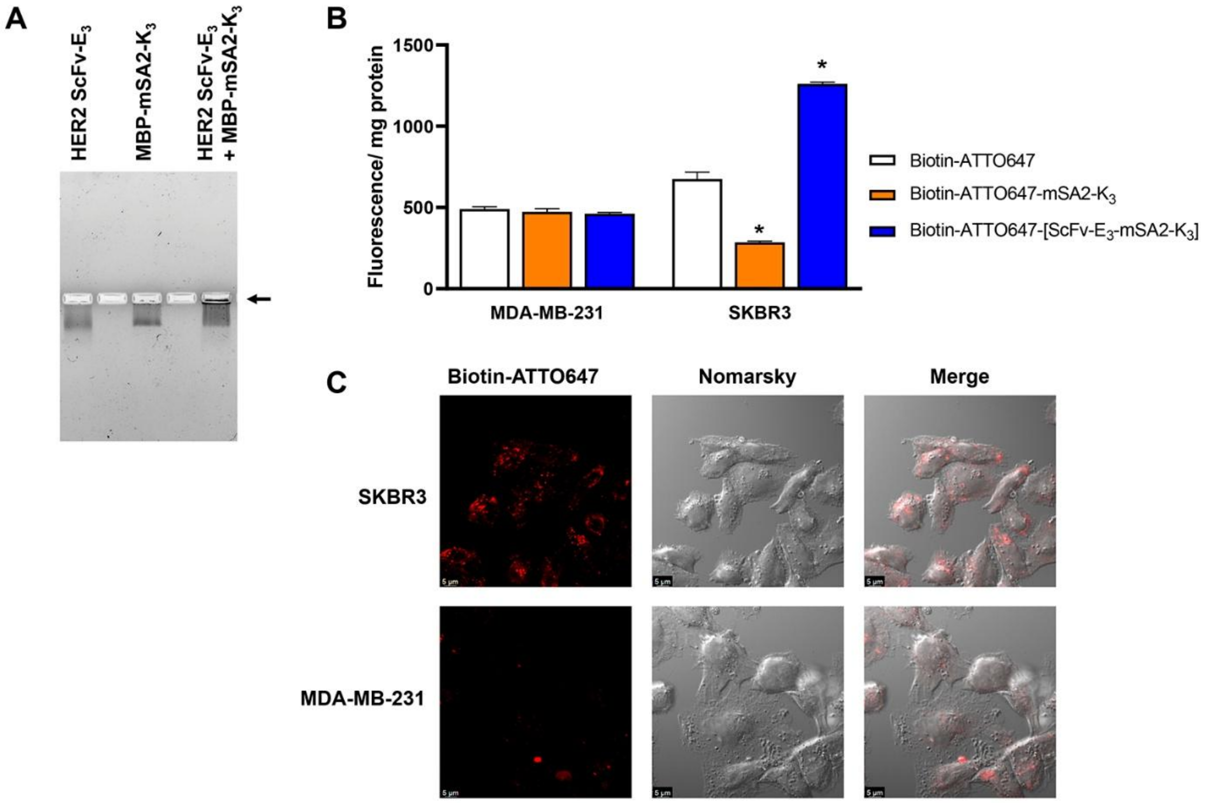

Supplement: Supplementary file 1 [file bm6c00502_si_001.pdf]
